# The Adenylyl Cyclase Plays a Regulatory Role in the Morphogenetic Switch from Vegetative to Pathogenic Lifestyle of *Fusarium graminearum* on Wheat

**DOI:** 10.1371/journal.pone.0091135

**Published:** 2014-03-06

**Authors:** Jörg Bormann, Marike Johanne Boenisch, Elena Brückner, Demet Firat, Wilhelm Schäfer

**Affiliations:** Biocenter Klein Flottbek - Department of Molecular Phytopathology and Genetics, University of Hamburg, Hamburg, Germany; University of Nebraska-Lincoln, United States of America

## Abstract

Cyclic 3′,5′-adenosine monophosphate (cAMP) is a nucleotide derived from adenosine triphosphate that acts as a second messenger throughout all kingdoms. Intracellular cAMP levels are synthesized by a membrane-bound protein, the adenylyl cyclase. In order to analyze the function of this gene and the importance of cAMP in the life cycle of the cereal pathogen *Fusarium graminearum*, the adenylyl cyclase gene (FGSG_01234) was deleted by gene replacement (Δ*Fgac1*). The Δ*Fgac1* mutant displayed a drastically reduced growth on agar medium which could be rescued by a cAMP analogon. Furthermore, the Δ*Fgac1* mutant was unable to produce perithecia on detached wheat nodes. However, artificial conditions like carrot agar allowed perithecia development. Pathogenicity towards wheat was drastically reduced in Δ*Fgac1* compared to the wild type. Point-inoculated spikelets showed only small lesions but no typical head blight disease symptoms. Fluorescence microscopy using dsRed-expressing strains revealed that the Δ*Fgac1* strain was unable to develop any complex infection structures like lobate appressoria and infection cushions. Instead, hyphal anastomosis occurs frequently. Scanning electron microscopy demonstrated the lack of fungal penetration. Hence, the formation of compound appressoria seems to be essential for infection of wheat. Hyphae on flower leaves produced huge amounts of new conidia, thereby circumventing the infection cycle. This abundant sporulation on wheat epidermis was not observed in wild type. Intriguingly, the *Fgac1* deletion mutant was able to infect maize cobs as wild type, indicating that cAMP signaling is not important for maize infection. The Δ*Fgac1* mutant was unable to produce the mycotoxin deoxynivalenol both *in vitro* and during wheat infection. In this study, we show that cAMP signaling controls important cellular processes such as development of infection structures, pathogenicity, secondary metabolite production and sexual reproduction. For the first time, we show that cAMP regulates the switch from vegetative to pathogenic lifestyle of *F. graminearum* on wheat.

## Introduction

*Fusarium graminearum* is the main causal agent of Fusarium head blight (FHB) disease of small grain cereals like wheat (*Triticum aestivum*) and barley (*Hordeum vulgare*) and of ear rot of maize. Full virulence on wheat relies on the production of the highly toxic trichothecene deoxynivalenol (DON). It is synthesized by the fungus in high amounts during infection but also post harvest. Its production is prerequisite for the colonization of the rachis that connects the florets within a wheat spike. However, for the initial penetration, DON biosynthesis is dispensable, although its biosynthesis is enhanced during formation of compound appressoria on the surface of wheat floral organs [1]. This raises the question which signals trigger the mycotoxin biosynthesis and the development of infection structures and how these signals are perceived.

In general, sensing of environmental cues, the transduction of signals and an appropriate intracellular response are essential for the evolutionary success of all organisms. A key role in signal transduction cascades falls to second messengers which are small molecules like cyclic nucleotides (cAMP and cGMP), hydrogen peroxide (H_2_O_2_), 1,2-diacylglycerol (DAG), inositol 1,4,5-trisphosphate (IP_3_) or ions like calcium (Ca^2+^). Second messengers act as transducers for information from multiple upstream signals and may activate multiple downstream targets (reviewed in [2]). Among these targets we find protein kinase cascades and transcriptional regulators. Together, the signal transduction cascade reaches from the signal perception by transmembrane receptors to a modulation in gene transcription.

In this paper, we highlight the importance of the cyclic adenosine monophosphate (cAMP) signaling cascade for the development and differentiation of the cereal pathogen *F. graminearum*. The cAMP signaling network is presumably best characterized in mammals (reviewed in [3]) and yeasts, i.e. *Schizzosaccharomyces pombe* [4], [5] and *Saccharomyces cerevisiae* [6], [7]. Briefly, an extracellular signal is perceived and transmitted to the intracellular space by membrane associated receptors. Attached to these receptors are heterotrimeric G-proteins consisting of three subunits named α, β, and γ. Upon activation, this complex dissociates into the α-monomer and a β-γ-dimer. The α-subunit is supposed to activate a membrane bound enzyme, the adenylyl cyclase, leading to the conversion of ATP to cAMP and pyrophosphate. Cyclic AMP acts on the regulatory subunits of the cAMP-dependent protein kinase A (PKA). Upon binding of cAMP, these subunits dissociate and give rise to active catalytic PKA-subunits. In this state, the PKA is able to activate a plethora of downstream effectors (summarized e.g. in [8]). In phytopathogenic fungi like *Magnaporthe oryzae* and *Ustilago maydis*, cAMP signaling is an important trigger for pathogenesis (reviewed in [9], [10]). Phytopathogenic fungi evolved distinct strategies for host penetration. Hyphae of the hemi-biotrophic grass pathogen *Claviceps purpurea* for example undergo no obvious morphological change prior invasion [11]. Penetration of the plant surface is enzyme dependent, since the deletion of two endopolygalacturonase genes leads to almost apathogenicity [12]. Other phytopathogenic fungi form infection structures that are, in general, regarded as modified hyphae specialized on the invasion of plant tissues. Best described are the classical appressoria of *M. oryzae*. A classical appressorium is known as a dome-shaped swollen cell at the tip of the conidial germ tube. A mature classical appressorium is separated from the germ tube by a septum and darkly pigmented by melanized cell walls. Melanized cell walls can stand a high turgor pressure, which is built up to perforate the host cell wall by a so-called penetration peg or hyphae [13]. Another type of appressoria are the so-called compound appressoria. Compound appressoria include multicellular lobate appressoria and infection cushions. Both types are formed by epiphytic mycelium and, usually, not by germ tubes (reviewed in [14], [15]).

In *Botrytis cinerea*, disruption of the cAMP-signaling cascade, namely the adenylyl cyclase [16], one catalytic subunit, and the regulatory subunit of the PKA, evoked a drastic reduction in vegetative and pathogenic development. Interestingly, however, pseudoappressoria were still formed and, thus, the initial penetration of the plant surface was only slightly delayed [17]. Adenylyl cyclase deficient mutants of *M. oryzae* fail to penetrate the plant. Their ability to form appressoria is abolished [18]. Thus, intracellular cAMP levels contribute to pathogenic development, although adenylyl cyclase activity seems not to be equally crucial for infection structure development in all necrotrophs. *F. graminearum* is regarded as a hemibiotrophic pathogen at least in early stages of infection, i.e. during plant surface penetration and initial intercellular growth. In later stages, however, a switch in the lifestyle towards necrotrophy can be observed (reviewed in [19]). Microscopic analysis using dissected wheat floret organs revealed that *F. graminearum* holds a large arsenal of infection structures of increasing complexity [1]. Initially, the plant cuticle is penetrated by simple infection hyphae emerging as side-branches from long, mostly unbranched runner hyphae. After colonization of the surface, hyphal branching becomes more frequent and infection structures of higher complexity appear: lobate appressoria and infection cushions. Underneath these complex structures, numerous penetration pores are detectable [1].

In this study we provide evidence for a critical role of cAMP-related signaling in the initiation of a pathogenic interaction of *F. graminearum* with wheat. Disruption of cAMP signaling by deletion of the *F. graminearum* adenylyl cyclase gene blocks infection structure development and completely impedes penetration of wheat epidermal cells, instead vegetative reproduction is induced. Furthermore, we provide data on the impact of cAMP-signaling on sexual reproduction and DON biosynthesis.

## Materials and Methods

### Fungal Strains and Culture Conditions

All mutants described in this study derived from the *F. graminearum* wild-type strain PH1. For vector cloning, the uracil-auxotrophic *S. cerevisiae* strain FGSC 9721 (FY 834) was used. Plasmids were amplified using *Escherichia coli* strain DH5α. Bacteria were cultivated in sterile lysogeny broth (LB) medium [20] either as a liquid culture or on agar plates. Yeast cells were cultured in YPG and in SD medium lacking uracil.

Conidiation of *F. graminearum* was induced in liquid wheat medium (15 g wheat leafs, shredded and autoclaved in 1 l H_2_O) incubated for seven days at 28°C. Sexual reproduction was monitored on carrot agar plates and detached wheat nodes placed on water agar plates (double autoclaved wheat parts with a nodium in the middle). Conidia of the wild type (WT:PH1), the *Fgac1* deletion strain (Δ*Fgac1*), the ectopic (ECT), and the complemented strain (c*Fgac1*) were inoculated on carrot agar plates and incubated at 28°C in the dark. After 3 days postinoculation (dpi), the aerial mycelia were knocked down with 1 ml of sterile 2.5% Tween 60 solution, using a sterile glass rod. The carrot agar plates and wheat nodes on water agar plates were further incubated at 18°C under near-UV light and white light with a 12-h photoperiod for up to 8 weeks. To assay ascospore viability, the lids of petri dishes containing mature perithecia were covered with thin layer of CM. Forcibly discharged ascospores that landed on the CM gave rise to new colonies which were then documented. For growth assays, mycelial plugs of the wild type and all mutant strains were taken from the edge of a 3-day-old colony on complete medium (CM; [21]) and placed in the middle of the assay plates. Supplements for the growth assays are indicated in the figure legends. All plates were incubated at 28°C for at least 3 days in the dark. The diameter of the colonies was measured using a technical ruler. The analyses were performed with at least 5 replicates.

### Vector Construction for Deletion and Complementation of *Fgac1*

Standard recombinant DNA methods were performed according to Ausubel *et al.* [22] and Sambrook *et al.* [20]. The open reading frame of *Fgac1* was disrupted using a double homologous recombination approach. For vector construction, flanking regions (720 kb upstream and 504 kb downstream of *Fgac1*) were cloned by PCR from genomic DNA (gDNA) using the primers listed in table S1. The PCR was initiated by denaturation at 94°C for 4 min, followed by 35 cycles of 94°C for 45 s, 55°C for 45 s and 72°C for 60 s. The PCR included a final extension step at 72°C for 10 min and a cooling step at 4°C. The replacement vector was constructed using yeast recombinational cloning method (YRC; [23]). For the replacement construct, the gene flanks and a cassette comprising the gene encoding a hygromycin phosphotransferase driven by the glycerinaldehyd-3-phosphate dehydrogenase promoter (P*gpd*) from *A. nidulans* together with the linearized pRS426 plasmid comprising a ampicillin resistance cassette and a gene facilitating uracil biosynthesis were co-transformed into yeast strain FGSC 9721 (FY 834). Homologues overhangs to adjacent fragments facilitate recombination and fusion of all fragments to give rise to a circular plasmid that confers uracil prototrophy. Prototrophic clones were selected and checked by PCR (data not shown). Plasmids were isolated from PCR-positive clones and transformed into *E. coli*. The replacement fragment was released from the plasmid (named pRS426:delta*Fgac1*) by restriction with *Bam*HI and *Kpn*I and used for fungal transformation. The complementation construct (pRS426:c*Fgac1*) was also generated by use of the YRC technique. Using a proof-reading polymerase, the entire ORF (7245 bp) and 1672 bp of upstream sequence were amplified in five parts (primers 9–18; see table S1) with overlapping sequence to the adjacent fragments. These fragments were co-transformed into the yeast strain FGSC 9721 (FY 834) together with a nourseothricin resistance cassette and a fragment of 1129 bp (primer 19 and 20) with homology to the 3′-UTR of the adenylyl cyclase gene. Plasmid characterization and isolation were performed as described above.

For the fungal transformation (see below), the complementation construct was excised from the plasmid using *Bae*I and *Sac*II. The vector pII99::dsRed was used for generation of dsRed-fluorescent strains [24]. This vector facilitates the constitutive expression of dsRed under the control of the *gpdA* promoter of *A. nidulans* and confers resistance to geneticin.

### Transformation of *F. graminearum*

A solution of 30–50 µl containing approximately 10 µg DNA was used for protoplast transformation of *F. graminearum*. The protoplast transformation method was performed as described previously [25], [26]. 100 ml of YEPD medium (0.3% yeast extract, 1% bacto peptone, 2% D-glucose) was inoculated with 1×10^6^ conidia and incubated overnight at 28°C, 150 rpm. The mycelia were collected by filtering with a 200 µm-diameter sieve and washed with double-distilled water. Mycelia were resuspended in a 20 ml mixture of driselase and lysing enzymes (Life Technologies, Darmstadt, Germany; 2.5%: 0.5% in 0.6 M KCl) and incubated for 2–3 h at 30°C, 80 rpm. Undigested hyphal material was removed from the protoplast suspension by filtration. The protoplasts were pelleted by centrifugation at 670×*g*, washed once with 10 ml STC (20% sucrose, 10 mM Tris-HCl, pH 8.0, 50 mM CaCl_2_), centrifuged again, then resuspended and adjusted in STC at 1×10^8^ protoplasts per ml. For transformation, 200 µl of the protoplast suspension was mixed with DNA. The samples were incubated at room temperature for 20 min. Subsequently, 1 ml PEG (40% polyethylene glycol 4000, 60% STC) was added and again incubated at room temperature for 20 min. The protoplast suspension was added to 5 ml TB3 medium (100 g sucrose, 0.3% yeast extract, 0.3% casamino acids) and shaken overnight at room temperature and 100 rpm for cell wall regeneration. The regenerated protoplasts were pelleted by centrifugation at 4.200×*g*, and then mixed with TB3 agar (1.5%) at 50°C with hygromycin B (250 µg ml^−1^). The mixture was then plated out on petridishes (10 ml/plate). After 24 h, an overlay comprising of TB3 agar (1.5%) and 500 µg ml^−1^ hygromycin B was added to the plates. Putative transformants were obtained after 2 dpi at 28°C. They were transferred to fresh plates of CM supplemented with 250 µg/ml hygromycin B and incubated at 28°C. The transformants were purified by single-spore isolation and subsequently checked by diagnostic PCR (fig. S1, table S1).

### Southern Blot Analysis

For southern hybridization analysis, approximately 3 µg of genomic DNA of the wild type, the Δ*Fgac1* and ectopic mutant strains was restricted with *Hin*dIII and *Dra*I, respectively overnight (fig. S1). The digested DNA was then separated on 0.8% agarose gels by electrophoresis at 70 V for 6–7 h. Then, the DNA was transferred by capillary blotting onto Hybond NX membranes (GE Healthcare, Munich, Germany), then hybridized with DIG (digoxygenin)-labelled (Roche, Penzberg, Germany) DNA probes. Detection and visualization procedures were carried out following the manufacturer’s manual (Roche).

### Virulence Assays on Wheat and Maize

The maize inbred line A188 [27] was grown in the greenhouse (temperature: 26°C−30°C, humidity: 70%–85%, natural daily photoperiod with additional artificial light when required). Before inoculation, the silks were manually pollinated to ensure optimal pollination. Each maize cob was inoculated by injecting conidia suspensions into the silk channel of primary ears using a syringe and cannula [28]. The infection procedure was performed with 2 ml of conidial suspension at a concentration of 2×10^4^ conidia ml^−1^ of the wild type and the Δ*Fgac1* mutant strains, respectively. Maize cobs inoculated with 2 ml of pure water were used for the negative control. The inoculated cobs were enclosed in plastic bags for the first 3 days and the inoculation lasted for 5 weeks. Infection assays were repeated 20 times for each strain.

The susceptible spring wheat cultivar Nandu (Lochow-Petkus, Bergen-Wohlde, Germany) was used for wheat virulence assays. Plants were cultivated in a growth room at 20°C with a photoperiod of 16 h and 60% relative humidity, then transferred to infection chambers with optimized conditions. A suspension of 500 conidia in 10 µl of the wild type and all mutants was inoculated into each of two central spikelets at the early stages of anthesis [29]. The inoculated spikes were enclosed in small plastic bags misted with water for the first 3 days, and then monitored for up to three weeks in the infection chambers. Wheat spikes inoculated with 10 µl pure water were used as the negative control. Wheat infection assays were repeated 30 times for each strain.

For the bioassay with detached floral leafs, paleas, and glumes were detached from the floret with a razor blade and placed in Petri dishes containing 1.6% (w/v) agar in water. The adaxial side of glumes and paleas were inoculated with 5 µl sterile water containing 20 conidia µl^−1^. Glumes which were used for electron microscopy were washed with 0.01% (v/v) Tween 20 for 10 min and afterwards rinsed twice with sterile H_2_O prior to inoculation. The washing step was included to remove wheat pollen from the glume surface.

### Deoxynivalenol (DON) Production Analysis

For *in-vitro* DON production measurement, 5×10^3^ conidia of the wild type, and the *Fgac1* mutant strains were pre-cultured in YPD for 3 days. The mycelia were harvested by filtering with a 200 µm diameter sieve, washed by rinsing at least three times with double-distilled water and dried on sterile Whatman paper (GE Healthcare, Munich, Germany). Then, 1 g of semi-dried mycelia was further incubated for 7 days in minimal medium supplemented with 5 mM γ-amino butyric acid (GABA) for DON induction. 50 µl of each supernatant was taken for DON measurement using a highly sensitive DON ELISA technique (RIDAscreen DON kits, R-Biopharm AG, Darmstadt, Germany). The DON amount was later normalized to the fungal dry weight. For the *in-planta* analysis, four wheat spikes were each inoculated with the wild type and the mutant strains, respectively. The spikes were inoculated with pure water as the negative control. The inoculated samples were collected after 7 dpi and 200 mg were dried in vacuum and ground to a powder under liquid nitrogen. Then, 50 mg of ground material was suspended in 500 µl of distilled water. The extract was manually mixed by vortex and centrifuged. For the DON quantification assay 50 µl of the supernatant was used. The production of DON was measured following the manufacturer’s instructions. All *in-planta* measurements were subsequently normalized to the amount of mycelium in the sample using quantitative PCR, as previously described [30].

### Quantification of cAMP

Intracellular cAMP concentrations were determined using a highly sensitive ELISA (Cyclic AMP EIA Kit, Cayman Chemical, Ann Arbor, MI, U.S.A). Wheat heads (21 dpi) and maize kernels (35 dpi) inoculated with the wild type, the Δ*Fgac1* mutant and water, respectively, were used for analysis. Samples were harvested, shock frozen in liquid nitrogen, lyophilized and ground in liquid nitrogen using a Retsch mill (Retsch, Haan, Germany). 50 mg of powder was extracted in 250 µl of 5% (w/v) trichloroacetic acid by vortex. Cell debris was pelleted by centrifugation and the supernatants were used in several dilutions (1∶5, 1∶10, and 1∶100) for the assay.

### Microscopic Analysis

Overview studies of inoculated floral organs of wheat were done with the MZFLIII fluorescence stereomicroscope (Leica Microsystems, Heerbrugg, Switzerland). Fungal development and stages of infection as described by [1] were monitored daily up to 3 weeks. The dsRed fluorescence of all dsRed expressing reporter strains used was detected with the Leica dsRed filter set containing an excitation filter at 546/12 nm and a long pass filter at 560 nm. Inclined reflected light of an external halogen lamp KL 1500 Electronic (Schott, Mainz, Germany) was used to visualize plant necroses as well as the mycelium under normal light conditions. Infection structures of the reporter strains were investigated by fluorescence microscopy using Zeiss Axio Imager.Z1 microscope equipped with a Zeiss Apotome (Zeiss, Oberkochen, Germany). A UV (ultra violet) lamp HAL 100 served as UV light source. DsRed was excited in the range of 538 to 562 nm and detected in the 570 to 640 nm range. The plant apoplast was excited in the range of 335 to 383 nm, while the blue autofluorescence was detected in the 420 to 470 nm range. Images were taken with Zeiss AxioCam MRm CCD camera. Image processing, including overlay of different fluorescence channels and generation of maximum intensity projections (MIP) of z-stacks was done with Zeiss AxioVision software (version 4.8.1). The epidermal and subepidermal invasion of wheat paleas inoculated with dsRed-expressing strains of the wild type and the Δ*Fgac1* mutant were studied in tissue cross sections by laser scanning microscopy (LSM). For LSM, a Zeiss LSM 780 microscope was used. DsRed was excited at 561 nm and the fluorescence detected at 570–640 nm. The plant apoplast was illuminated at 405 nm and its blue autofluorescence detected at 410–490 nm. Image processing of z-stacks was performed with Zeiss ZEN software (version 2010). For scanning electron microscopy, inoculated floret tissues of wheat were fixed according to Huang *et al.* [31] with 4% (v/v) glutaraldehyde in 50 mM phosphate buffer (pH 6.8) for 8–10 h at 4°C, then rinsed with the same buffer for 3 h. Afterwards, samples were post-fixed with 1% (w/v) osmium tetroxide in the same buffer for 2 h at 4°C. After dehydration of samples for 24 h by a graded acetone series at room temperature, they were critical-point dried using a CPD 030 SCD 050 (BAL-TEC, Pfäffikon, Switzerland). Dried samples were mounted on stubs with Ponal Classic (Henkel, Düsseldorf, Germany) placed on carbon tabs. After 48 h in a desiccator samples were sputter-coated with gold using a SCD 050 (BAL-TEC). The scanning electron microscope SEM LEO 1525 Zeiss) was used operating at 6 kV. To identify penetration pores in the epidermis of inoculated glumes, the mycelium, including infection structures, was removed from the plant surface. The removal was performed with critical point dried samples by using an adhesive tape (TESA, Hamburg, Germany). Afterwards the sample was mounted on stubs and sputter-coated as described above.

## Results

### Generation of Mutants

A comparative database survey using known orthologues of adenylyl cyclases led to the identification of the putative *F. graminearum* orthologue, FGSG_01234. Functional domain analysis using the Conserved Domain Database on NCBI [32] revealed the presence of a G-alpha binding domain, a mononucleotidyl cyclases domain, and two regions containing leucine-rich repeats for protein-protein interactions. In order to functionally characterize this protein, we deleted most of the ORF by targeted gene replacement. For this purpose, flanks comprising sequence of the 5-prime UTR and the 3-prime end of the gene were fused to a hygromycin resistance cassette and transformed into the *F. graminearum* wild type isolate PH1 (fig. S1A). Among the 51 primary transformants we obtained, only one showed an integration of the construct at the adenylyl cyclase locus. However, repetitive single-spore isolations were necessary to obtain a homokaryotic deletion strain. This mutant (Δ*Fgac1*) was confirmed by PCR and Southern blot analysis (fig. S1B and C) and subsequently used for transformation with plasmid pII99::dsRed, conferring the red-fluorescent protein dsRed to be constitutively expressed in the cytosol. An analogous wild-type mutant was also generated. In order to univocally prove that the observed phenotypes are due to the deletion of the adenylyl cyclase gene, we reintroduced the gene and approximately 1000 kb upstream sequence to complement the mutant.

### Deletion of *Fgac1* has Dramatic Impact on Vegetative Growth and Conidia Production

**Figure 1:**
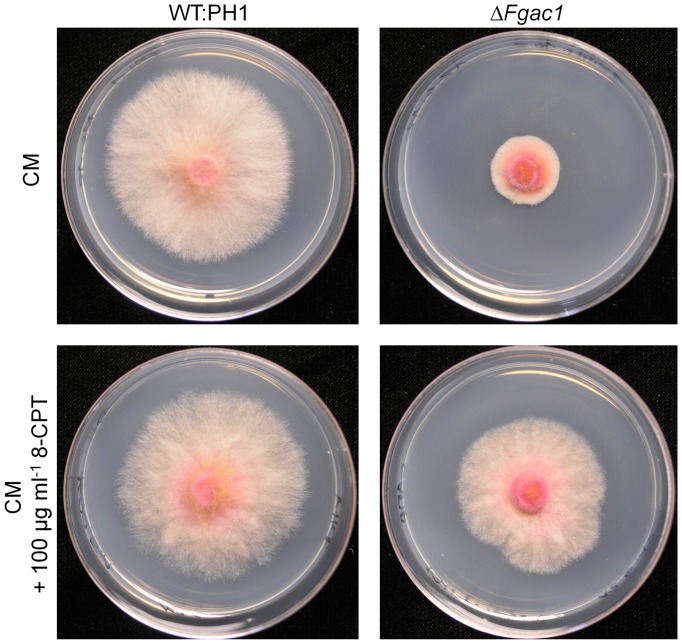
Colony morphology of the wild type (WT:PH1) and the Δ*Fgac1* on complete medium (CM) and CM supplemented with the cAMP analogon 8-(4-Chlorophenylthio)adenosine 3',5'-cyclic monophosphate sodium salt (8-CPT). Addition of 8-CPT partially complements the growth phenotype.

In an agar plate assay, vegetative growth of the Δ*Fgac1* mutant was dramatically reduced compared to the wild type and the complemented strain on CM (fig. 1 and fig. S2). In *N. crassa*, the growth rate of adenylyl cyclase deficient mutants varies according to the carbon source that is supplemented [33]. The reduced growth rate of the Δ*Fgac1* mutant in contrast, was independent of the carbon source. Growth of the wild type and the complemented strain were indistinguishable (fig. S2). Addition of the 8-substituted cAMP derivative 8-(4-Chlorophenylthio)adenosine 3',5'-cyclic monophosphate sodium salt (8-CPT) partially restituted the colony morphology and growth rate of the deletion strain on CM, indicating that a lack of cAMP is responsible for the growth defect (fig. 1).

*Fusarium graminearum* is able to propagate through the production of large quantities of asexual spores. Sporulation can be induced in liquid medium containing extract from wheat leaves. In this medium, the wild type and complemented strain produced over 6 times more conidia than the Δ*Fgac1* mutant within one week (fig. S3). On solid medium, in contrast, the deletion mutant displayed a hypersporulation phenotype. While the wild type did not sporulate, the dense colonies of the Δ*Fgac1* mutant produced 10.93±1.73, 18.75±2.05, 24.5±1.96, and 14.6±0.77 conidia×µl^−1^ on carrot agar, wheat decoction agar, maize broth agar, and CM agar, respectively. The germination rates of conidia from all strains were similar and close to 100% (data not shown).

### Disruption of cAMP Signaling Abolishes DON Biosynthesis *in vitro* and *in planta*

**Figure 2:**
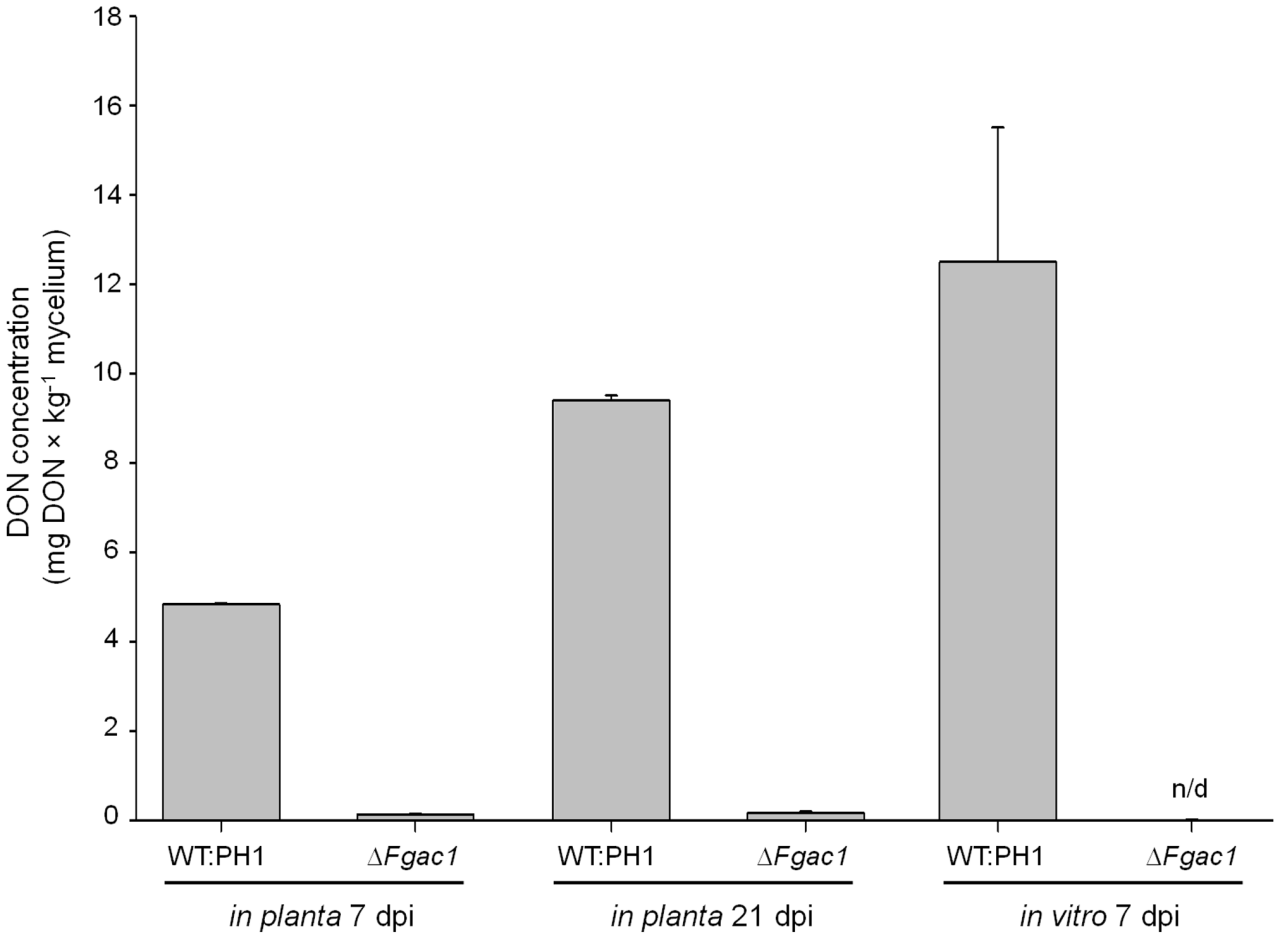
DON concentrations in wheat heads and under *in-vitro* induction conditions. For the wild type (WT:PH1) and the Δ*Fgac1* mutant the DON concentration was determined by ELISA. *In-planta* measurements were conducted after 7 and 21 days postinoculation (dpi). The *in-vitro* DON-production was assayed after 7 dpi in medium containing 5 mM gamma amino butyric acid. Under all condition, the level of DON produced by the Δ*Fgac1* mutant was below the detection limit of the ELISA. Toxin measurements were repeated twice with four replicates each. DON amounts were normalized to the amount of fungal material by qPCR (*in planta*) and dry weight (*in vitro*), respectively. Error bars indicate the standard deviation.

*Fusarium graminearum* accumulates considerable amounts of DON during wheat head infection and when grown in liquid medium containing γ-amino butyric acid (GABA) as sole nitrogen source (J. Bönnighausen and W. Schäfer, unpublished results). While the wild type accumulated 125.0 (±30.1) mg×kg^−1^ DON, the supernatants of the mutant were free of DON at 7 dpi in induction media. All measured amounts were far below detection limit (fig. 2). Hence, cAMP is an essential trigger for DON biosynthesis *in vitro*. To check whether cAMP is also necessary for DON production during infection, we inoculated heads of the susceptible wheat cultivar Nandu with conidia of the wild type and the deletion strain and monitored DON accumulation after 7 and 21 dpi. Intriguingly, even after 21 dpi, the mutant did not produce detectable amounts of DON. The wild type, in contrast, synthesized 4.83 (±0.03) mg×kg^−1^ and 9.4 (±0.11) mg×kg^−1^ DON after 7 and 21 dpi, respectively (fig. 2). The *in-vitro* DON measurements were normalized to the fungal dry mass, while the *in-planta* DON measurements were normalized to the amount of fungal biomass using qPCR as described previously [30]. Taken together, these results demonstrate that DON biosynthesis in culture as well as during wheat infection depends on cAMP signaling.

### The *F. graminearum* Adenylyl Cyclase is Required for Perithecia Development on Wheat Straw

**Figure 3:**
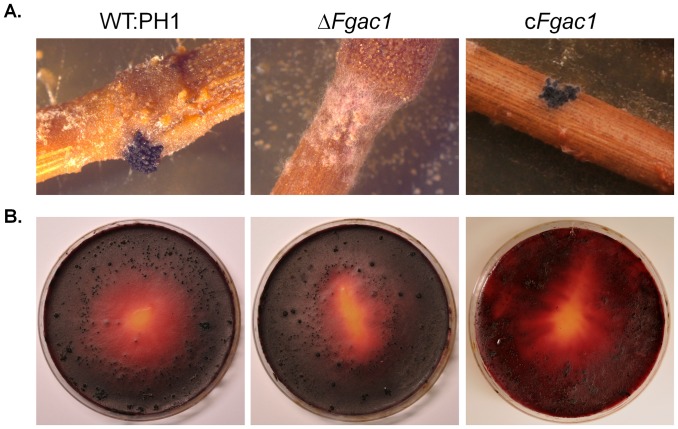
Perithecia development on detached wheat nodes (A) and carrot agar (B). The wild type (WT:PH1), the Δ*Fgac1*, and the complemented mutant were assayed. Clusters of perithecia developed on both substrates when inoculated with the wild type and the complemented mutant within 3 weeks. The mutant produced immature perithecia clusters on wheat nodes and wild-type like clusters on carrot agar.

The ability of *F. graminearum* wild type and the *Fgac1* deletion mutant to produce perithecia was assayed on two substrates: on wheat straw and on carrot agar. When inoculated on autoclaved wheat straw, the wild type usually forms clusters of perithecia within four weeks. On carrot agar plates it takes approximately three weeks. Deletion of the adenylyl cyclase in *F. graminearum* leads to mutants unable to differentiate perithecia on wheat straw but forming normal appressoria on carrot agar. Within four weeks the mutant produced large clusters of perithecia on carrot agar (fig.3B). On wheat straw, in contrast, the mutant only formed aggregates of hyphae similar to protoperithecia. However, even after prolonged incubation, these protoperithecia never mature (fig. 3A). Hence, cAMP signaling might be involved in surface or substrate recognition that promotes sexual reproduction. Reintroduction of the adenylyl cyclase to the mutant complemented the phenotype (fig. 3). To test whether the perithecia produced by the deletion mutant on carrot agar contained ascospores that are forcibly discharged and viable, the lids of petri dishes containing mature perithecia were covered with CM agar as described previously [34]. Ascospores that land on the medium should give rise to new colonies. Above wild-type and deletion mutant perithecia, newly formed colonies were frequently observed (fig. S4).

### A Functional cAMP-signaling Cascade is Essential for Infection Structure Development

In a previous study we demonstrated that *F. graminearum* forms specialized infection structures on the surface of inoculated floral organs of wheat [1]. In order of appearance and increasing complexity these infection structures comprise simple foot structures (infection stage I), lobate appressoria, and infection cushions (summarized as compound appressoria; infection stage II). All infection structures develop from epiphytically growing runner hyphae.

We investigated the ability of the Δ*Fgac1* mutant to differentiate from runner hyphae to complex infection structures. For this purpose, paleas and glumes were dissected from florets and inoculated with conidia of dsRed-expressing strains deduced from the wild type (named WT-dsRed) and the Δ*Fgac1* mutant (named Δ*Fgac1*-dsRed).

**Figure 4:**
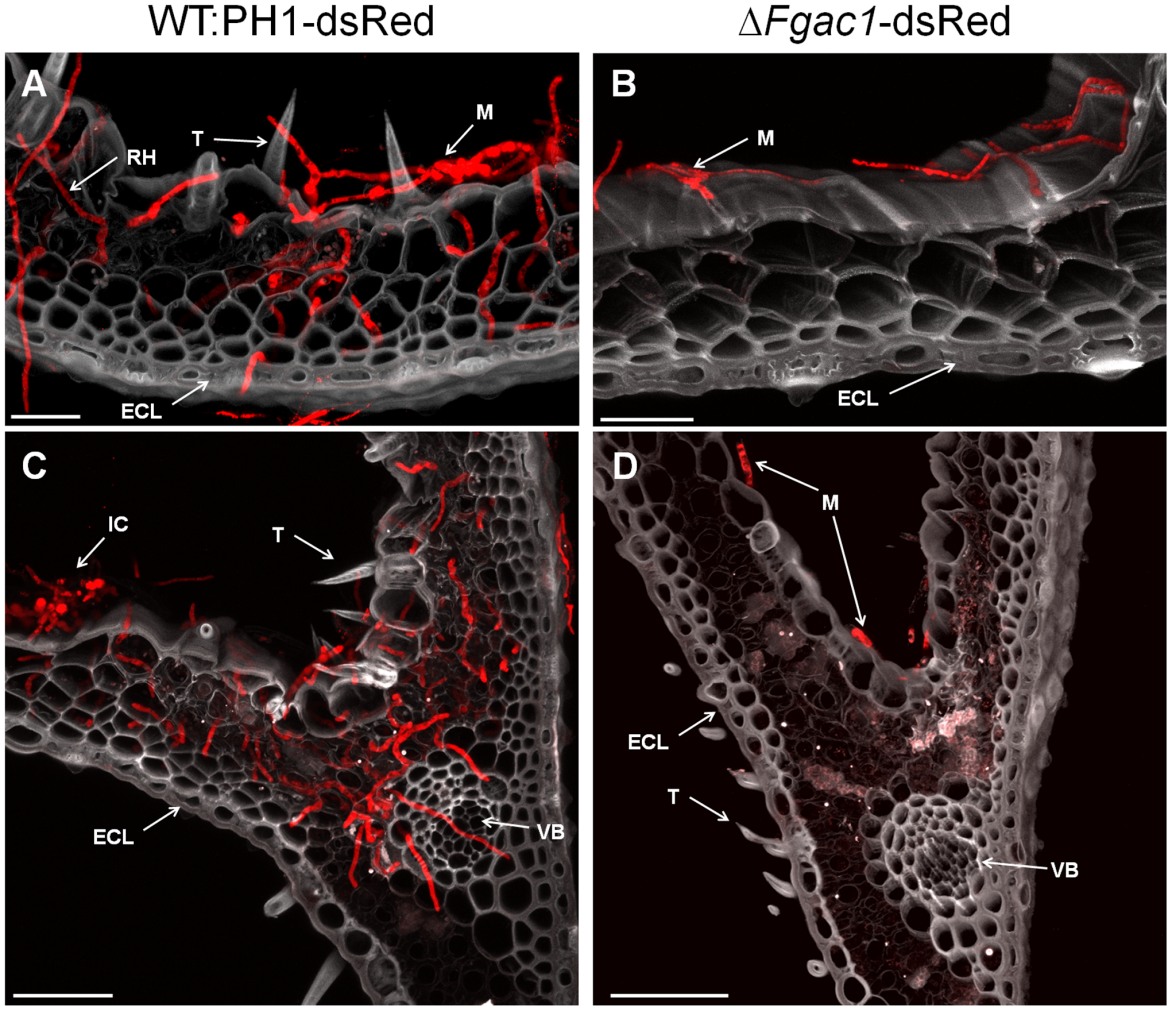
Infection assay on dissected wheat paleas. A–D. CLSM micrographs of cross-sections at 9 (**A; B**) and 8 days postinoculation (dpi) (**C; D**). Paleas were inoculated with dsRed-expressing mutants derived from the wild type (WT:PH1) and the Δ*Fgac1*-mutant. The wild type penetrated the epidermal cell layer (ECL) and colonized the inner cells of the palea (**A; C**). The Δ*Fgac1*-mutant, in contrast, only grew superficially and never penetrated the ECL (**B; D**). In grey is the auto-fluorescence of the plant. A diffuse autofluorescence around the vascular bundles in (**D**) indicates a putative plant response. Other abbreviations: IC, infection cushion; M, mycelia; RH, runner hyphae; T, trichome; VB, vascular bundles. Scale bar: 20 µm.

**Figure 5:**
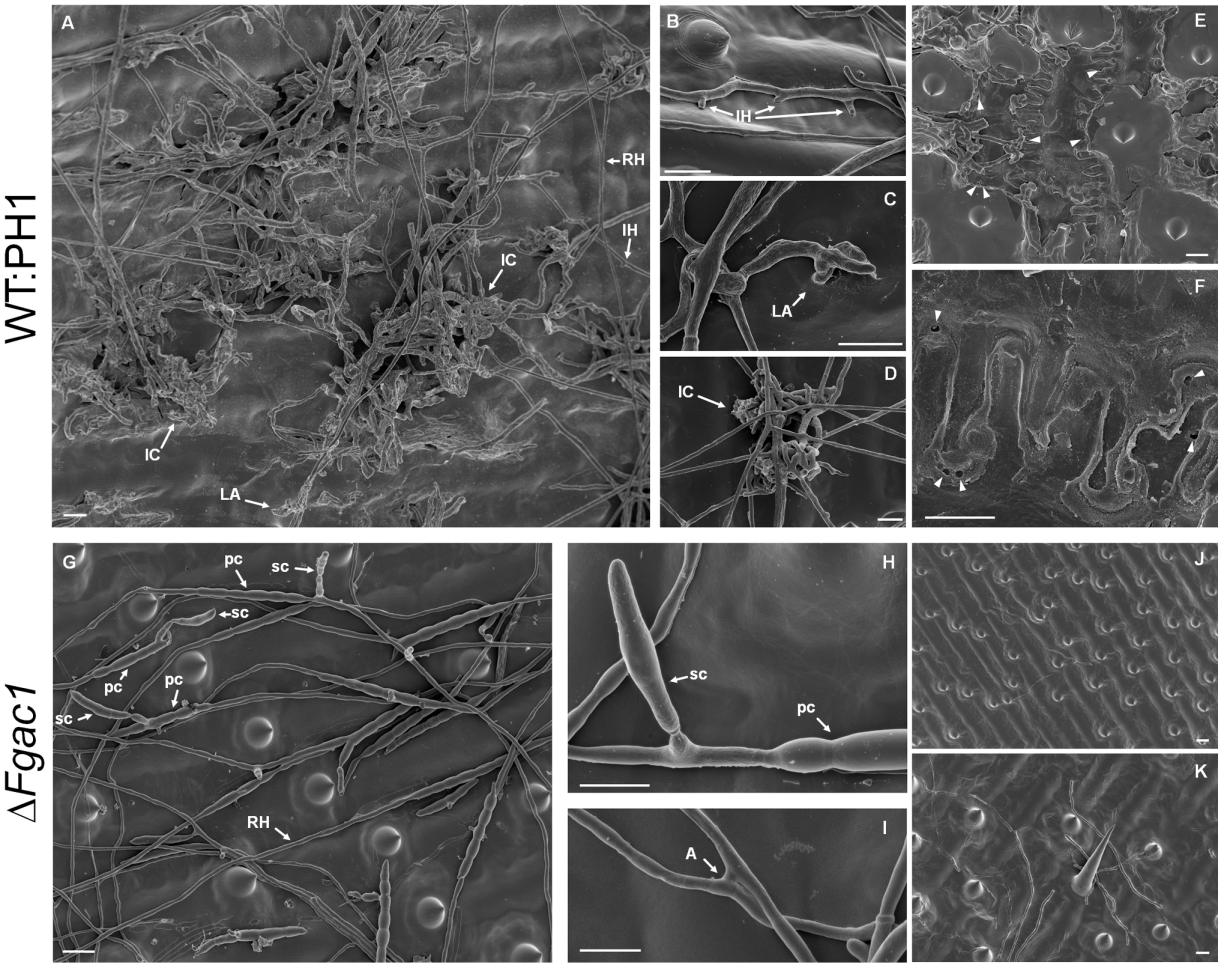
Infection assay on dissected wheat glumes. Scanning electron micrographs showing the WT:PH1 (**A–F**) and the Δ*Fgac1*-mutant (**G–K**) on the surface of a glume at 8 days postinoculation (dpi). **A.** Overview picture of the WT:PH1 producing infection structures. **B–D.** Detailed micrographs of three kinds of infection structures produced by the wild type: infection hyphae (**B**), lobate appressoria (**C**), and infection cushions (**D**). **E–F.** The plant surface after removal of infection cushions using an adhesive tape. The epidermal plant cell wall below infection cushions show several penetration pores (arrowheads) of about 1 µm in diameter. **G–K.** The mutant failed to develop any kind of infection structures. Instead, it grew in straight hyphae (runner hyphae, RH) that rarely formed side branches. Furthermore, it produced masses of conidia (**G**). **H.** A germinating primary conidium (pc) immediately gave rise to a secondary conidium (sc). **I.** The mutant hyphae formed anastomoses between epiphytic runner hyphae. **J–K.** The plant surface after removal of epiphytically growing hyphae. The epidermal plant cell wall appears intact. Abbreviations: IC, infection cushion; LA, lobate appresorium; IH, infection hyphae; A, anastmosis. Scale bar: 10 µm.

WT-dsRed displayed a vigorous growth on the surface of the palea (fig. 4A) after 8 dpi. Starting at 9 dpi, infection cushions were observed (fig. 4C). In the Δ*Fgac1* mutant, germination of conidia and colonization of the surface of paleas by runner hyphae took place within 24 hpi (data not shown). However, in contrast to wild-type infections, development of compound appressoria was never observed in the mutant, even after prolonged incubation time (fig. 4B and D). Intriguingly, instead of complex infection structures, the mutant produced huge amounts of conidia and anastomoses (fig. 5G, H and I) starting at 4 dpi. Anastomoses between runner hyphae of the wild type were never observed. The mutant produced frequently new conidia immediately after germination of progenitor conidia (named secondary conidia (sc) in fig. 5G and H). Inoculation of palea with the Δ*Fgac1* mutant did not cause visible necrosis of plant tissue within 14 dpi. The wild type, in contrast, caused large-scale lesions after 14 dpi (fig. S5).

SEM and LSM analysis confirmed that type-I infection structures, such as infection hyphae, were absent in the mutant (fig. 4B, D and fig. 5G). Using SEM, a mucilage-like substance is visible between wild-type hyphae and the plant surface, especially in immediate vicinity of infection structures (fig. 5B and C). It is noteworthy that mucilage was never observed on samples inoculated with the Δ*Fgac1* mutant (fig. 5H and I).

In order to check for penetration events, mycelia of the wild type and the Δ*Fgac1* mutant were removed from glumes after 8 dpi. Underneath the mycelium of the Δ*Fgac1* mutant, we never detected penetration pores (Fig. 5J and K). The plant cuticle remained intact. Thus, the Δ*Fgac1* mutant’s hyphae do not facilitate successful colonization of the host plant. Underneath wild type infection structures, in contrast, we observed numerous penetration pores (fig. 5E and F). Further proof of the lack of penetration in the Δ*Fgac1* mutant is provided by data obtained from cross sections of paleas inoculated with the dsRed-expressing strains (fig. 4). Hyphae of WT-dsRed were visible inside epidermal cells, subepidermal parenchymya cells, and vascular cells at 8 dpi (fig. 4A and C). Δ*Fgac1*-dsRed never grew inside or beneath the epidermal cell layer (fig. 4B and D). Instead, we observed a diffuse plant-derived autofluorescence in parenchyma cells (fig. 4D). Since there is no hyphal growth detectable, a plant defense reaction is assumed. However, the nature of this reaction remains elusive. Addition of 8-CPT to the inoculum and 6 dpi facilitated plant penetration by the deletion mutant and, at least partially, restored the ability to form infection structures (fig. S6).

Taken together, these results point out that functional cAMP signaling is of vital importance for the pathogenic development of *F. graminearum*. The morphogenetic switch to conidia production at early stages of epiphytic growth reflects a tremendous disruption of the usual pathogenic circle in which conidia are produced only after a nearly complete decomposition of the host tissue.

### Cyclic AMP is a Host-specific Pathogenicity Factor

**Figure 6:**
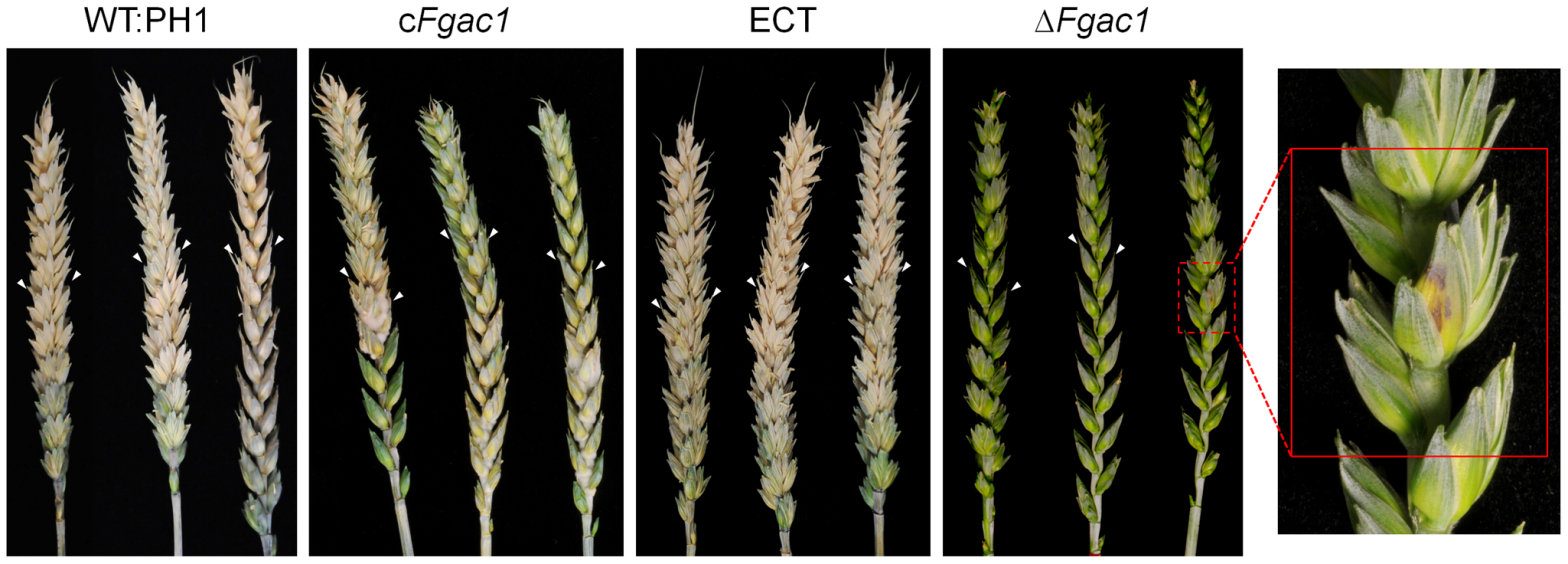
Pathogenicity assay on wheat. Wheat heads of the susceptible cultivar Nandu were inoculated with conidia of the wild type (WT:PH1), the Δ*Fgac1*, the complemented Δ*Fgac1* mutant (c*Fgac1*) and one ectopic mutant (ECT), respectively, and incubated 21 days. The Δ*Fgac1* mutant strain was drastically reduced in virulence. The inoculated spikelet showed necrotic lesions (close-up of the Δ*Fgac1*-inoculated spike). The wild-type, the c*Fgac1* and the ectopic strains caused symptoms typical for *Fusarium* head blight (FHB) disease. The wheat infections were performed 30 times for each strain.

Given the results described in the previous section, it was not surprising that the Δ*Fgac1* mutant turned out to be completely non-pathogenic on wheat. Wheat florets point-inoculated with conidia of the Δ*Fgac1* mutant displayed only minor lesions after 21 dpi while the wild type, the complemented and an ectopic mutant were fully infective and evoked typical Fusarium head blight symptoms (fig. 6). Addition of 8-CPT to the inoculum and 3 dpi could not restore the infectivity (data not shown). Maybe, a limited diffusibility and accessibility of 8-CPT in the inoculated wheat head might impede this assay.

**Figure 7:**
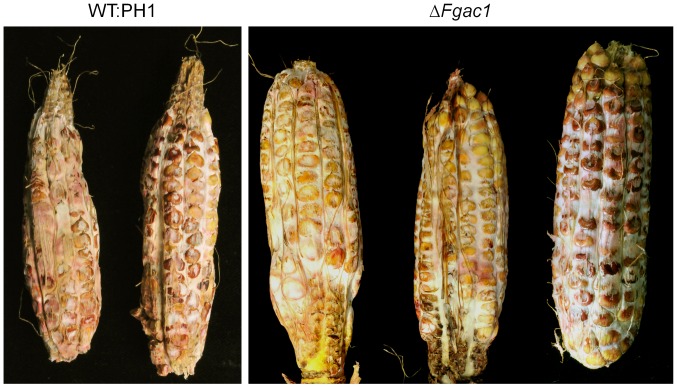
Pathogenicity assay on maize. Maize cobs of the inbreed line A188 were inoculated with conidia of the wild type (WT:PH1) and the Δ*Fgac1*, respectively, and incubated 35 days. The Δ*Fgac1* mutant strain and the wild type caused typical symptoms of cob rot disease. The maize infections were performed 20 times for each strain.

**Figure 8:**
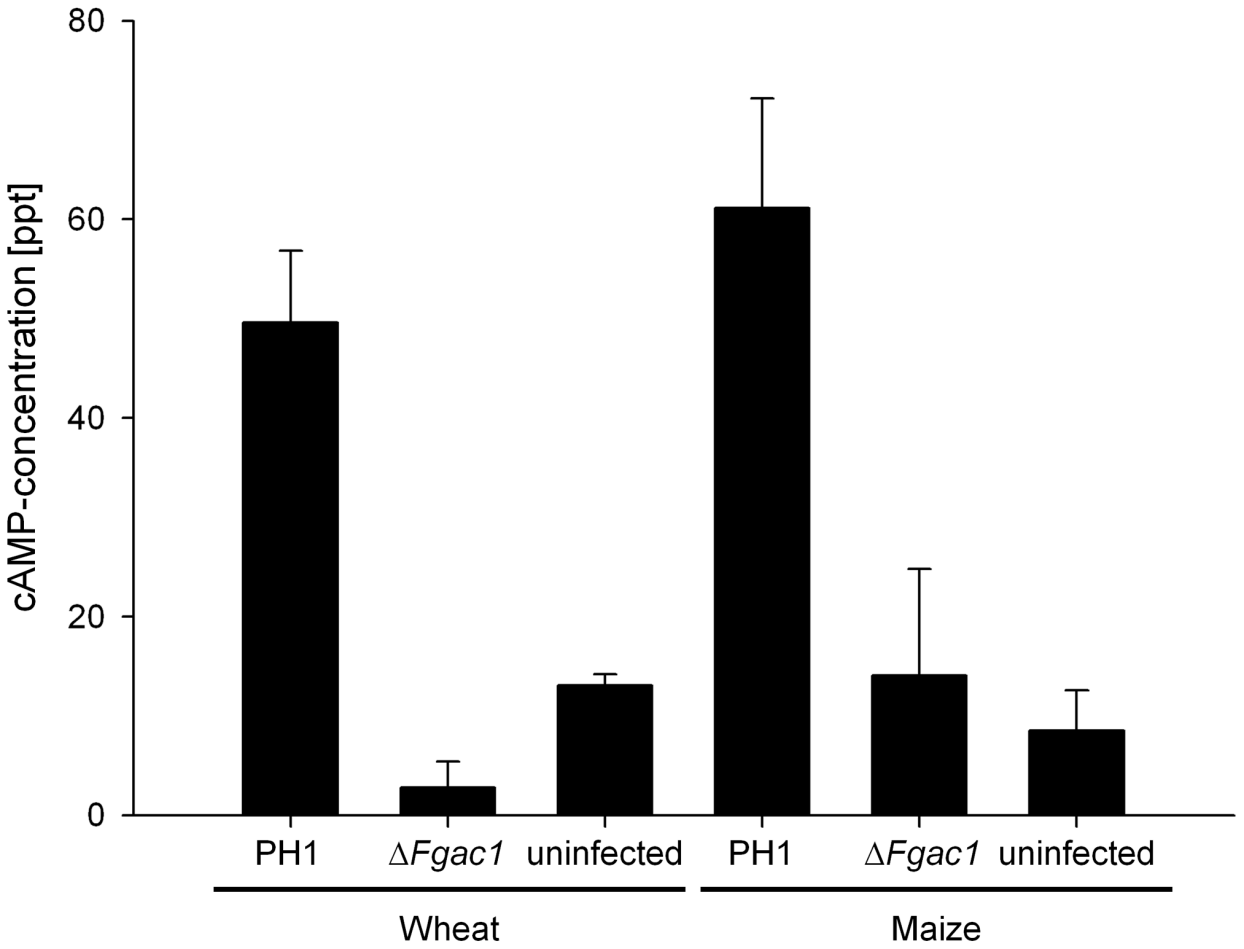
Measurement of cytosolic cAMP levels. Cyclic AMP specific enzyme-linked immunosorbent assay using samples obtained from wheat (21 days postinoculation (dpi)) and maize (35 dpi) inoculated with the wild type (WT:PH1), the Δ*Fgac1* mutant and water as a control. Error bars indicate the standard deviation. The cAMP measurement was done using two biological and three technical replicates each.

Unexpectedly, the mutant was fully virulent on maize (fig. 7). The maize inbred line A188 was inoculated with conidia of the wild type and the disrupted strain through the silk channel. After 35 dpi, the disease symptoms were evaluated. All maize cobs were colonized and the kernels were rotten and covered with reddish mycelium (fig. 7). There was no difference in symptom severity detectable between the mutant and the wild type. We tested whether or not corn had a high cAMP level that could rescue the defect of delta-Fgac1 mutant. Measurement of intracellular cAMP levels using ELISA did not reveal an accumulation of cAMP in uninfected and Δ*Fgac1*-mutant infected maize kernels. Only in wild-type infected maize kernels, cAMP levels increased significantly. Hence, cAMP signaling seems not to be necessary for maize infection. Since cAMP level do not increase in Δ*Fgac1* infected maize cobs we can deduce that nearly the entire cAMP derives from the fungus. The same pattern was observed in the wheat samples (fig. 8). The levels of cAMP were lower in the samples inoculated with the Δ*Fgac1*-mutant compared to the wild type and approximately the same when compared with the non-inoculated samples.

In summary, the results obtained in this study show the importance of cAMP in pathogenicity of *F. graminearum* on wheat. Impressively, we identified circumstances, under which this ubiquitous second messenger might be dispensable, i.e. infection of maize, conidia production on flower leaves, and perithecia development on artificial substrates.

## Discussion

The entire life cycle of *F. graminearum* in nature relies on the successful colonization of a susceptible host plant by its infecting agents, i.e. macroconidia and ascospores. Each step in the life cycle is triggered by environmental cues like temperature, light and humidity (reviewed in [35], [36]). Perception and subsequent processing of signals from a variable environment is based on a cascade comprising of receptors and intracellular modules that are able to transmit and amplify a signal to downstream effectors. Second messengers play a decisive role in these cascades. Here, we analyzed the ubiquitous second messenger cAMP for its function in virulence, vegetative growth, secondary metabolism, and sexual reproduction.

### Deoxynivalenol Biosynthesis is Controlled by cAMP

A prevalent secondary metabolite of *F. graminearum* is DON. It is harmful to humans and animals and is, in contrast to ZEA, a virulence factor for the infection of wheat. DON biosynthesis can be induced *in vitro* by several additives to media such as polyamines [37], [38], H_2_O_2_ [39], and cobalt chloride [40]. Furthermore, its biosynthesis is influenced by ambient conditions like temperature [41], and pH [42]. The underlying regulation processes and signaling cascades are complex and not fully revealed. Our results indicate that cAMP signaling cascade represent a superior regulatory framework for DON biosynthesis, since the *Fgac1* deletion mutants were deficient in DON biosynthesis both *in planta* and *in vitro*. The *in-planta* results are of limited impact, as the mutant was not able to penetrate and colonize the plant. Due to this lack of invasive growth, the areas of high DON induction within the wheat floret, i.e. the caryopsis and the rachis node [24], were never reached by the mutant. Recent studies *in F. graminearum* and *F. fujikuroi* demonstrate a complicated interplay between the different enzymes within the cAMP signaling cascade. Deletion of one (but not the two other) G alpha (named GzGPA1) and of the unique G beta subunit in *F. graminearum* resulted in an increased DON and ZEA biosynthesis *in vitro* [43]. None of the mutants however, was decreased in DON as was the Δ*Fgac1* mutant in our study. This suggests that, under DON inducing conditions, none of the G-protein subunits activate the adenylyl cyclase. It cannot be ruled out that functional redundancy in the G alpha subunits contribute to the minor phenotypes observed for single deletion mutants in the subunits compared to the deletion of the unique adenylyl cyclase. Deletion of the *F. fujikuroi* adenylyl cyclase, but not of the G alpha subunits, led to a down-regulation of gibberellic acid biosynthesis [44]. These results draw a picture of a complicated crosstalk between different regulators that act specifically in response to certain environmental cues (see also table S2).

### Disruption of Cyclic AMP Signaling Prevents Hyphal Differentiation Leading to the Formation of Infection Structures

Deletion of the *F. graminearum* adenylyl cyclase abolished the ability to develop compound appressoria. Intruigingly, the mutants were also unable to penetrate wheat epidermal cells. This correlation strongly suggests that the formation of complex infection structures is prerequisite for successful colonization of wheat tissue. On wheat floral organs, the mutant was hypersporulating which represents a dramatic morphogenetic switch in the lifestyle from pathogenic to vegetative growth. It seems plausible that the transition from runner hyphae to complex infection structures on wheat floral leaves is triggered by cAMP levels. Elevated cAMP levels in wild-type infected wheat samples, as proven by the ELISA analysis, may influence the formation of compound appressoria, while depletion of intracellular cAMP favors sporulation during wheat infection. Considering the wild-type like pathogencity of the Δ*Fgac1* mutant to maize, we have to assume that infection structures are less important for penetration of maize. Therefore, the pathogenic lifestyle occurs and early sporulation is suppressed. However, comprehensive histology of the infection process of *F. graminearum* on maize is still missing. Deletion of *tri5*, encoding a trichodien synthase in *F. graminearum*, drastically reduces virulence on wheat but has no influence on disease development on maize [45]. However, histological analysis of primary infection stages revealed that Δ*tri5* mutants are able to penetrate the host tissue in a wild-type like manner [1]. Hence, the pathogenicity phenotype in the Δ*Fgac1* mutant is most likely not correlated to the abolished DON biosynthesis.

It is proposed that both chemical and physical stimuli are involved in the induction of appressorium formation in *M. oryzae* [9], [18], [46] and that cAMP signaling is involved. Adenylyl cyclase deletion mutants of *M. oryzae* were unable to form appressoria on a hydrophobic surface and were non-pathogenic on rice leafs [18], [47], [48]. Instead, the mutants continue to grow as hyphae on the surface of inoculated onion epidermal tissue, a phenotype that we also observed in the Δ*Fgac1* mutant on glumes.

We, so far, did not identify conditions to induce compound appressoria formation *in vitro* on agar plates or artificial surfaces (M. J. Boenisch, unpublished results). Hence, specific and as-yet unknown compounds present on natural surfaces seem to be necessary for compound appressoria formation in *F. graminearum*. Exogenous nutrient sources such as plant exudates or sucrose generally promote an increase in branching frequency during infection cushion development of *R. solani* on artificial surfaces [49], [50]. Furthermore, the availability of nutrients is discussed to be necessary for the production of mucilage in *R. solani* [49] which might be necessary for adhesion of runner hyphae to surfaces [49]–[51] and thigmotropic growth which finally results in the formation of infection cushions. We also observed the formation of mucilage surrounding the wild-type, but not the mutant hyphae that colonize the host surface (fig.5, C, H and I). Hence, it is feasible that a defective sensing of a certain nutritional environment together with the absence of mucilage in the mutant strain contributes to the lack of infection structures.

### Disruption of cAMP Signaling Interferes with Sexual Reproduction, Conidiation and Hyphal Fusion

The lack of mature perithecia on wheat straw might be due to a dysfunctional sensing of the environment by the deletion mutant. Results from Krüger and co-workers [52] connect the cAMP signaling pathway with the regulation of pheromone responsive genes. Deletion of a G alpha subunit in *U. maydis* led to sterile mutants [52]. Deletion of one of three G alpha subunits (GzGPA1) in *F. graminearum* caused sterility on carrot agar plates [43]. Since the Δ*Fgac1* mutant was able to produce perithecia on carrot agar but not on wheat, it seems likely that different G alpha subunits contribute to the activation of the adenylyl cyclase, depending on the environmental conditions. It is tempting to speculate that a dysfunctional sensing of plant-derived nutrients or certain patterns on the plant surface lead to the sterility phenotype on wheat nodes. Carrot agar, in this regard, might contain substances that bypass the adenylyl cyclase pathway and, thereby, facilitate perithecia formation.

The observation that the mutant produces conidia during epiphytic growth on detached wheat floral leaves and on solid substrates like CM, but not in submersed culture, substantiates the hypothesis of an influence of nutrient sensing, since also conidia production is nutrient-dependent [53]. Interestingly, adenylyl cyclase knock-out mutants of *N. crassa* also sporulated under inappropriate conditions [54].

Also, a high abundance of hyphal fusion events (anastomoses) is regarded as an indicator for nutrient starvation conditions (see [55] and references therein). Accordingly, numerous anastomoses occur between runner hyphae of the Δ*Fgac1* mutant but not the wild type (fig. 5I). Hyphal fusions are believed to facilitate genetic exchange in a parasexual cycle, but can also be used to build up a hyphal network in order to acquire resources (reviewed in [56]). All these findings suggest that the Δ*Fgac1* mutant is not able to acquire nutrition on intact natural substrates.

**Figure 9:**
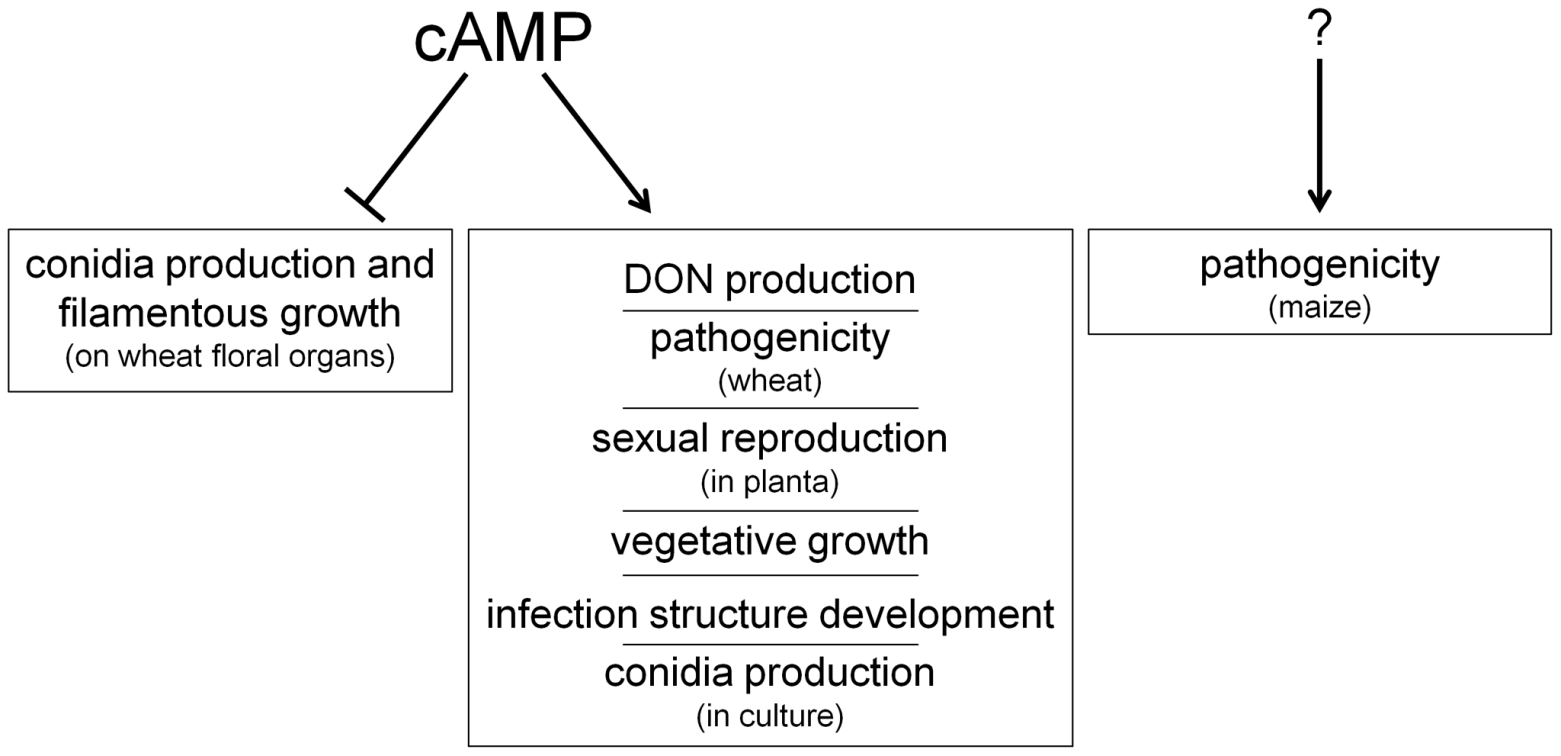
Proposed model: how FgAC1 influence numerous physiological functions like vegetative growth, sexual reproduction, DON biosynthesis, conidiation, and pathogenicity in *F. graminearum*. The results suggest that, at least on wheat, elevated cAMP levels favour pathogenic development in terms of infection structures and DON biosynthesis, while depletion of cAMP promotes conidiation and filamentous growth. Moreover, cAMP plays a role in the transduction of environmental cues, since sexual reproduction and conidiation phenotypes depend on growth conditions. Cyclic AMP is dispensable for maize infection. Here, other as-yet unknown factors support pathogenicity.

In the present study, we were able to shade some light on the complex regulation pattern that involves hyphal differentiation, DON production and virulence. Table S2 provides an overview on phenotypes observed after deletion of adenylyl cyclases in other phytopathogenic fungi. This compendium underscores the diverse role of cAMP in plant pathogens. While in *F. fujikuroi* a functional adenylyl cyclase is dispensable for virulence (at least against tomato fruits) it is mandatory for host invasion in *M. oryzae*, *C. neoformans* and others. Similarly, also other developmental processes like sexual and asexual propagation and secondary metabolism are affected to varying extents by cAMP depletion in different plant pathogens. In this study, it became obvious that cAMP is an essential second messenger for proper development of *F. graminearum* acting as a vital switch between vegetative growth and pathogenicity (summarized in fig. 9). Furthermore, we provide strong indications pointing towards a novel functional dependence of compound appressoria formation and wheat epidermis penetration by *F. graminearum*.

## Supporting Information

Figure S1 **Gene replacement, Southern hybridization and diagnostic PCRs for** ***Fgac1*** **. A.** Replacement and Southern hybridization strategy for *Fgac1*. Deletion of *Fgac1* (2) by homologous recombination using a replacement fragment excised from pRS426:delta*Fgac1* using restriction enzyme *Bam*HI and *Kpn*I (1) (3: genotype of disrupted strains). Flanking regions are indicated as bold black lines. The gene flanks were fused to a hygromycin resistance cassette, consisting of the resistance gene (hygromycin B phosphotransferase, *hph*), the *gpdA* promoter (P*-gpdA*), and *trpC* terminator (T*trpC*) *of A. nidulans*. Primer binding sites for PCR are indicated as small arrows (numbering refers to table S1). The regions used as probes for Southern analysis is represented by the dashed line. Scheme not to scale. **B.** Southern analysis of Δ*Fgac1* and the wild type. DNA of the mutant and wild type strain was digested using *Hin*dIII (for probe 1) and *Dra*I (for probe 2), separated on agarose gels, blotted on membranes and probed with a DIG-labelled probe for a fragment of the flanking region of *Fgac1* (probe 1) and for a gene-internal fragment of *Fgac1* (probe 2). Probe 1 hybridized with the DNA of the disruption mutant (6717 bps) and the wild type (2827 bps). Probe 2 only gave a signal in the wild type. **C.** PCR analysis of the Δ*Fgac1*, one ectopic, and three complemented mutants, and the wild type. Deletion of *Fgac1* was verified in one mutant (analyzed after single spore purification) using primers 5 and 6. The wild type, the ectopic strain (ECT) and the complemented mutants (c*Fgac1*) were PCR-positive for the gene internal fragment (915 bps).
(TIF)

Figure S2 **Colony morphology** of the wild type (WT:PH1), the Δ*Fgac1*, and the complemented mutant (c*Fgac1*) after 3 days of growth on minimal medium (MM) supplemented with different carbon sources and without any carbon source. The Δ*Fgac1* is strongly reduced in growth on all substrates tested when compared with the wild type and the complemented strain. Agar plates were inoculated with mycelial plugs from 3-day-old cultures.
(TIF)

Figure S3 **Conidia production assay.** Conidia were produced within 7 days in 65 ml liquid wheat medium inoculated with 10^4^ conidia of the wild type (WT:PH1), the Δ*Fgac1* and the c*Fgac1* strain, respectively. The assay was performed with three replicates each. The decrease in conidia production in the Δ*Fgac1* mutant is highly significant according to *t*-test (p<0.001).
(TIF)

Figure S4 Ascospore viability assay. To check if ascospores forcibly discharged by the strains were viable, the lids of petri dishes which carrot agar was inoculated with conidia of the wild type (WT:PH1) and the Δ*Fgac1* mutant, respectively, was covered with a thin layer of CM agar. Ascospores that land on the agar gave rise to new colonies indicating that they were viable.
(TIF)

Figure S5 **Assay for necrotic lesions development.** Dissected wheat paleas were inoculated with the Δ*Fgac1* mutant strain, the wild type and with water as negative control, respectively. The wild type evokes necrotic lesions after 5 days postinoculation (dpi). Paleas inoculated with the Δ*Fgac1* mutant strain and water, in contrast, remained symptomless within 14 dpi.
(TIF)

Figure S6 **Infection assay on dissected wheat paleas.** **A–D.** CLSM micrographs of cross-sections at 10 days postinoculation (dpi). Paleas were inoculated with a dsRed-expressing Δ*Fgac1*-mutant. 8-(4-Chlorophenylthio)adenosine 3',5'-cyclic monophosphate sodium salt (8-CPT) was added to the inoculum (**A; B**) and 6 dpi (**C; D**). The addition of 8-CPT restored the ability of the mutant to penetrate the ECL. In grey is the auto-fluorescence of the plant. Other abbreviations: IC, infection cushion; LA, lobate appressorium; M, mycelia; RH, runner hyphae; T, trichome. Scale bar: 20 µm.
(TIF)

Table S1 **Primers used in this study.**
(DOC)

Table S2 **Summary of phenotypes of adenylyl cyclase mutants in other plant fungal pathogens.**
(DOCX)
