# Supplementary figures and images for: The Adenylyl Cyclase Plays a Regulatory Role in the Morphogenetic Switch from Vegetative to Pathogenic Lifestyle of Fusarium graminearum on Wheat
Source: PLoS One. 2014 Mar 6;9(3):e91135. doi: 10.1371/journal.pone.0091135 (PMC3946419; doi:10.1371/journal.pone.0091135)

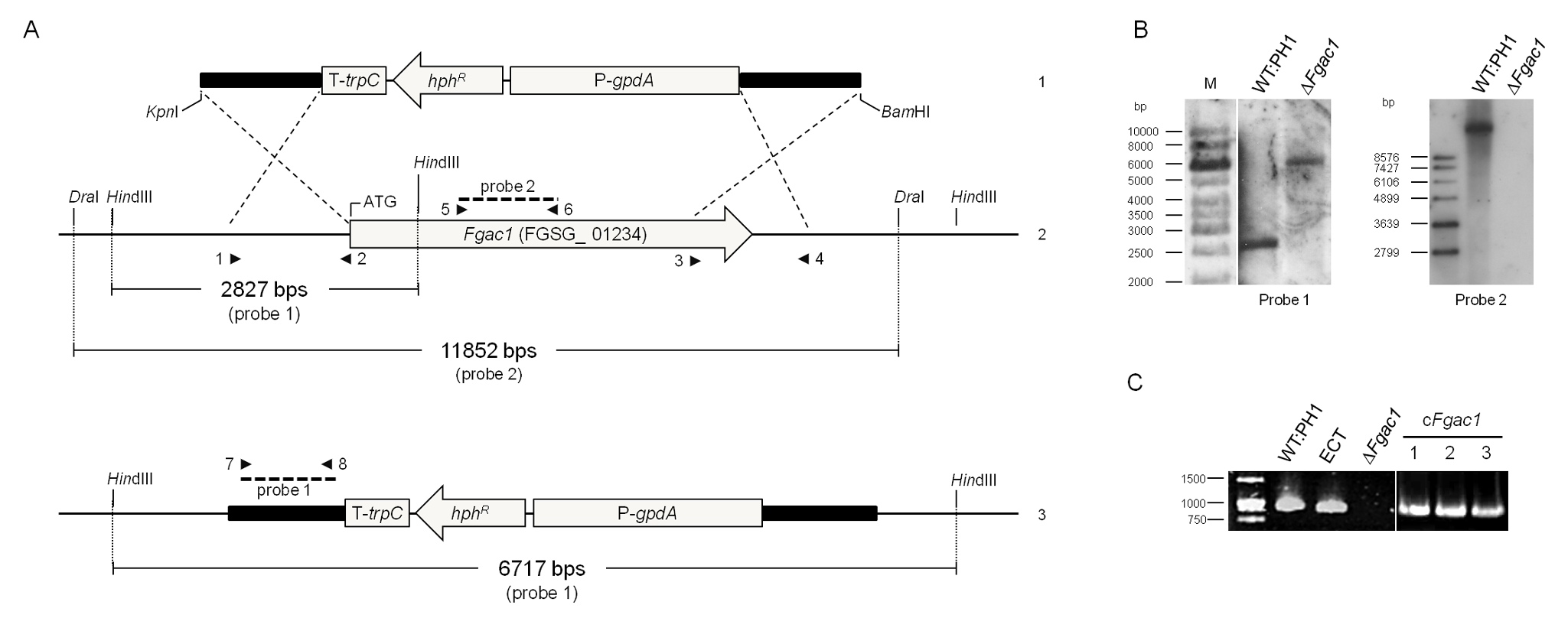

Supplement: Figure S1 — Gene replacement, Southern hybridization and diagnostic PCRs for Fgac1 . A. Replacement and Southern hybridization strategy for Fgac1. Deletion of Fgac1 (2) by homologous recombination using a replacement fragment excised from pRS426:deltaFgac1 using restriction enzyme BamHI and KpnI (1) (3: genotype of disrupted strains). Flanking regions are indicated as bold black lines. The gene flanks were fused to a hygromycin resistance cassette, consisting of the resistance gene (hygromycin B phosphotransferase, hph), the gpdA promoter (P-gpdA), and trpC terminator (TtrpC) of A. nidulans. Primer binding sites for PCR are indicated as small arrows (numbering refers to table S1). The regions used as probes for Southern analysis is represented by the dashed line. Scheme not to scale. B. Southern analysis of ΔFgac1 and the wild type. DNA of the mutant and wild type strain was digested using HindIII (for probe 1) and DraI (for probe 2), separated on agarose gels, blotted on membranes and probed with a DIG-labelled probe for a fragment of the flanking region of Fgac1 (probe 1) and for a gene-internal fragment of Fgac1 (probe 2). Probe 1 hybridized with the DNA of the disruption mutant (6717 bps) and the wild type (2827 bps). Probe 2 only gave a signal in the wild type. C. PCR analysis of the ΔFgac1, one ectopic, and three complemented mutants, and the wild type. Deletion of Fgac1 was verified in one mutant (analyzed after single spore purification) using primers 5 and 6. The wild type, the ectopic strain (ECT) and the complemented mutants (cFgac1) were PCR-positive for the gene internal fragment (915 bps). (TIF) [file pone.0091135.s001.tif]

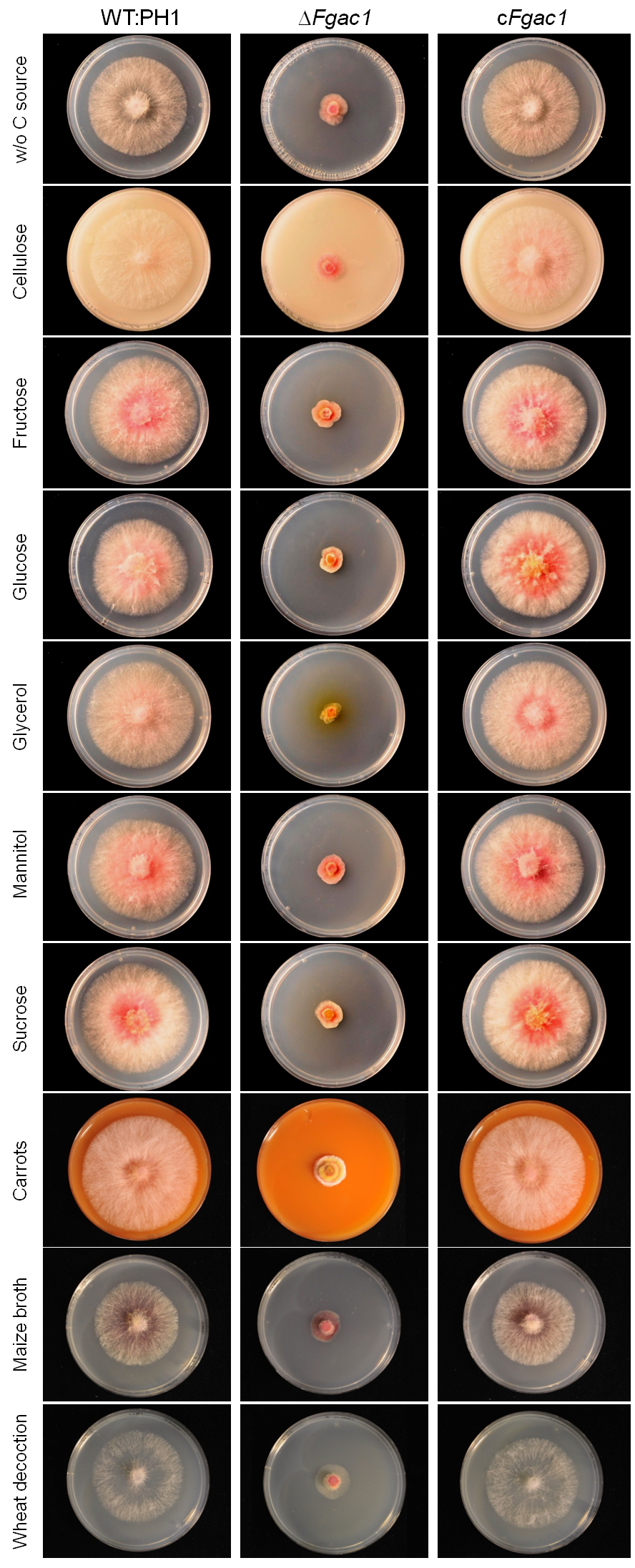

Supplement: Figure S2 — Colony morphology of the wild type (WT:PH1), the ΔFgac1, and the complemented mutant (cFgac1) after 3 days of growth on minimal medium (MM) supplemented with different carbon sources and without any carbon source. The ΔFgac1 is strongly reduced in growth on all substrates tested when compared with the wild type and the complemented strain. Agar plates were inoculated with mycelial plugs from 3-day-old cultures. (TIF) [file pone.0091135.s002.tif]

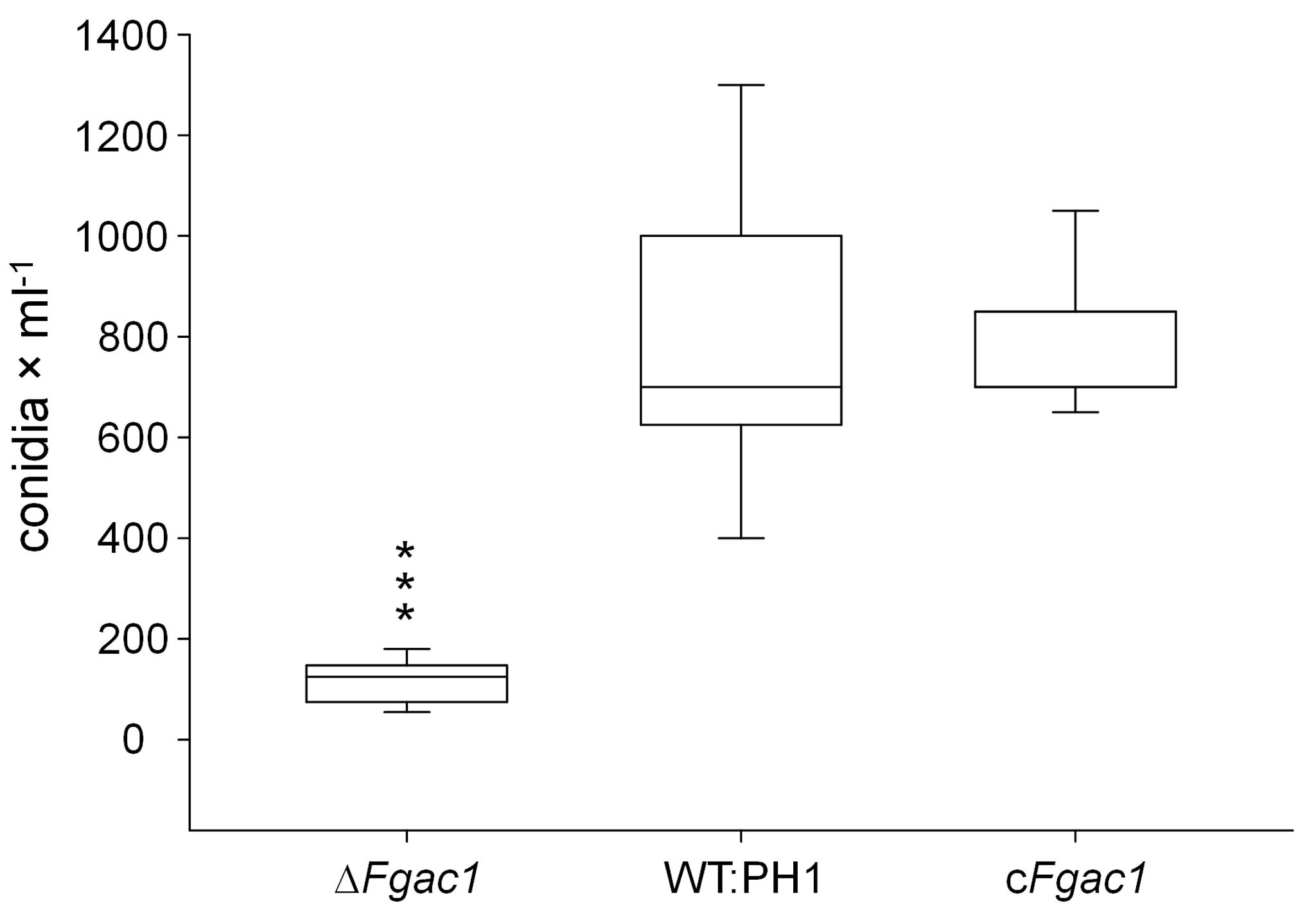

Supplement: Figure S3 — Conidia production assay. Conidia were produced within 7 days in 65 ml liquid wheat medium inoculated with 104 conidia of the wild type (WT:PH1), the ΔFgac1 and the cFgac1 strain, respectively. The assay was performed with three replicates each. The decrease in conidia production in the ΔFgac1 mutant is highly significant according to t-test (p<0.001). (TIF) [file pone.0091135.s003.tif]

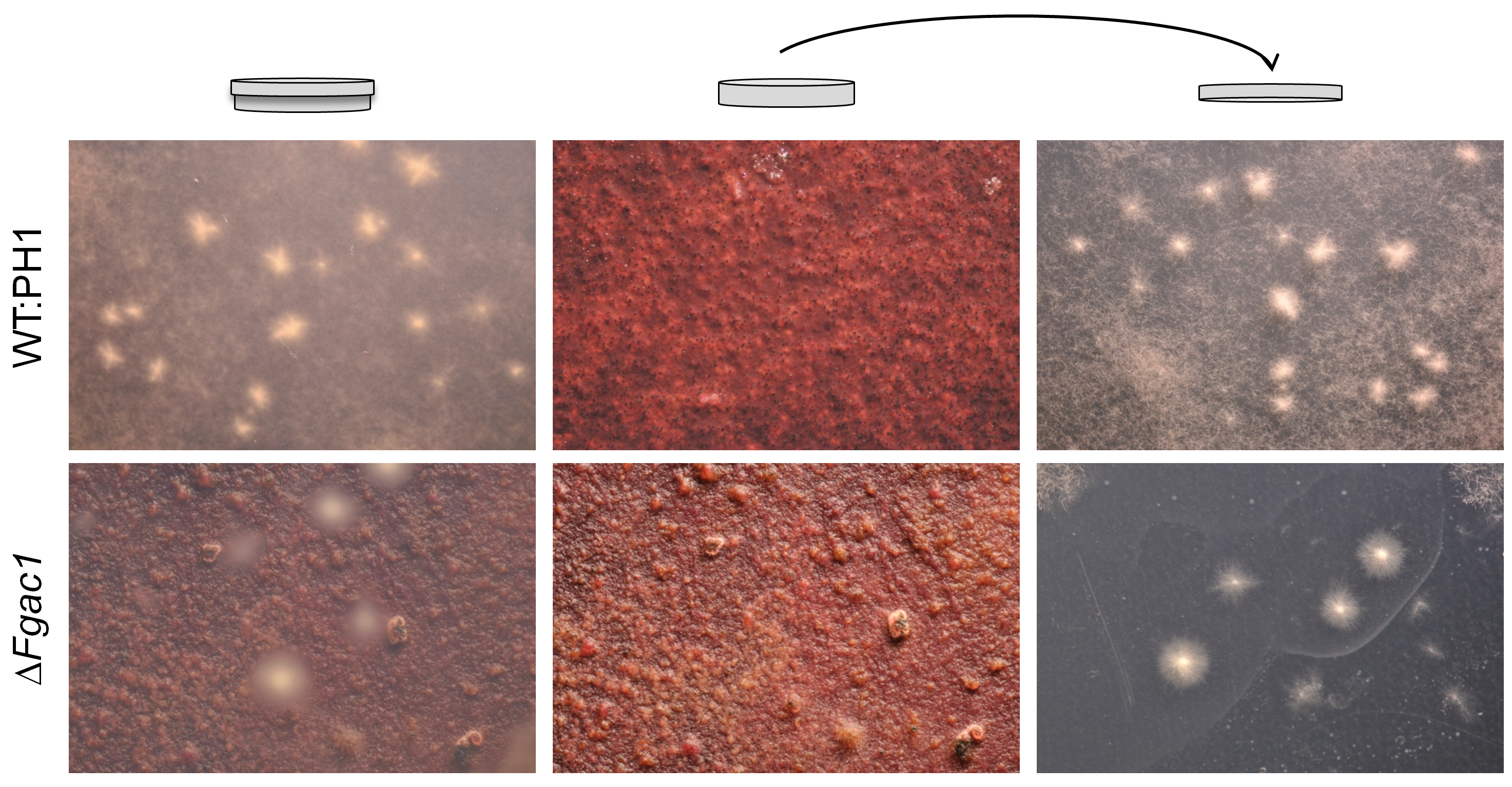

Supplement: Figure S4 — Ascospore viability assay. To check if ascospores forcibly discharged by the strains were viable, the lids of petri dishes which carrot agar was inoculated with conidia of the wild type (WT:PH1) and the ΔFgac1 mutant, respectively, was covered with a thin layer of CM agar. Ascospores that land on the agar gave rise to new colonies indicating that they were viable. (TIF) [file pone.0091135.s004.tif]

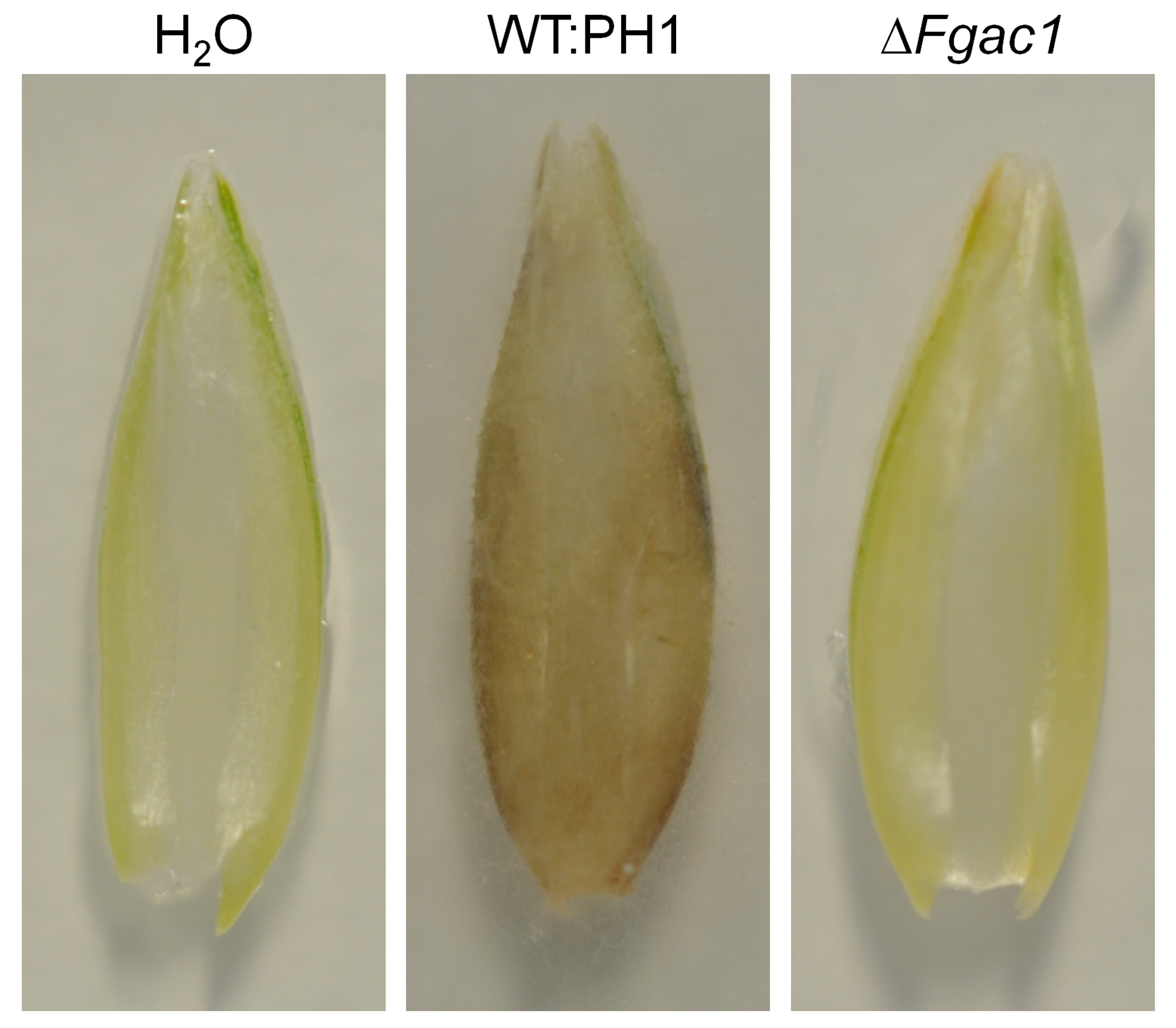

Supplement: Figure S5 — Assay for necrotic lesions development. Dissected wheat paleas were inoculated with the ΔFgac1 mutant strain, the wild type and with water as negative control, respectively. The wild type evokes necrotic lesions after 5 days postinoculation (dpi). Paleas inoculated with the ΔFgac1 mutant strain and water, in contrast, remained symptomless within 14 dpi. (TIF) [file pone.0091135.s005.tif]

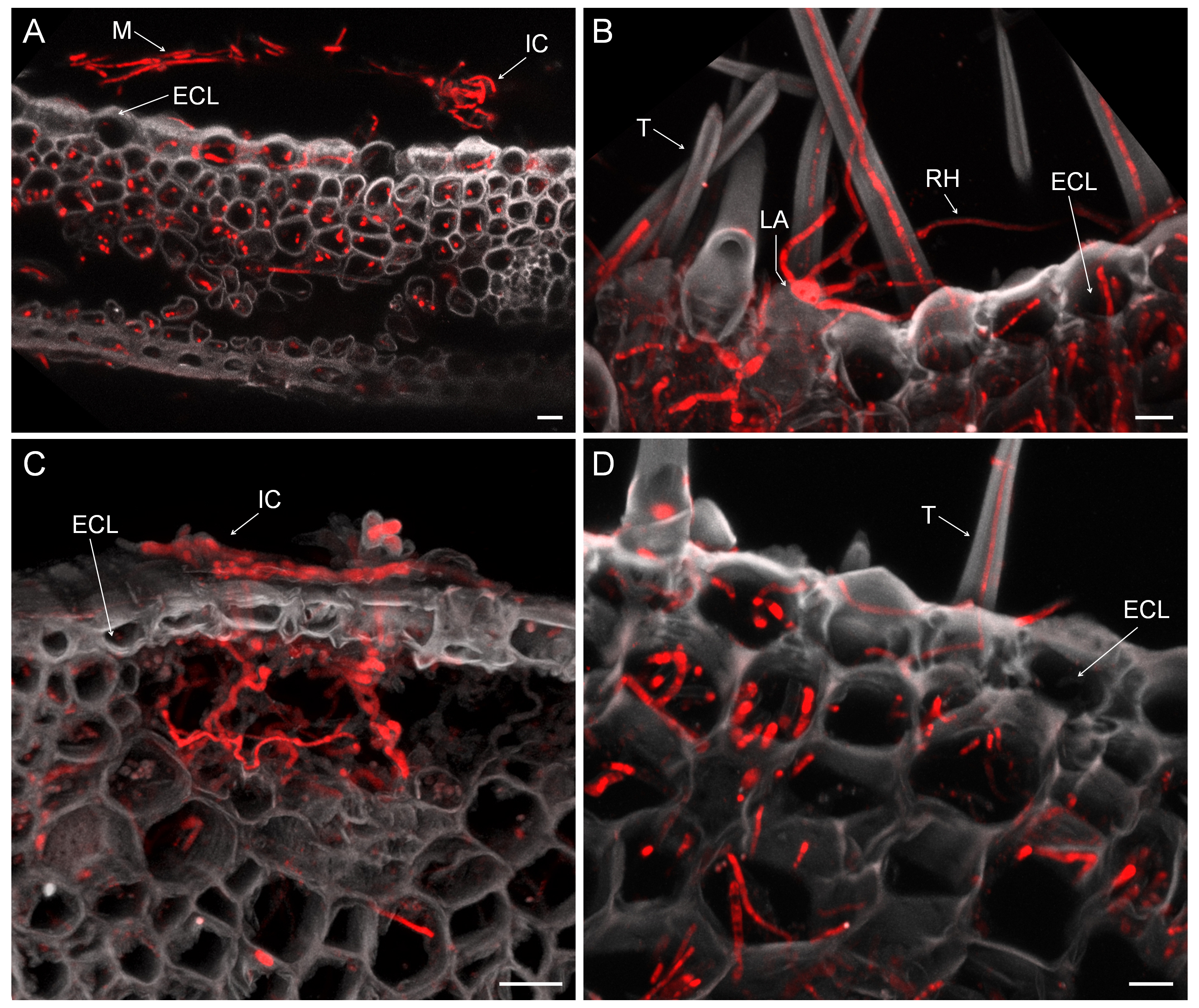

Supplement: Figure S6 — Infection assay on dissected wheat paleas. A–D. CLSM micrographs of cross-sections at 10 days postinoculation (dpi). Paleas were inoculated with a dsRed-expressing ΔFgac1-mutant. 8-(4-Chlorophenylthio)adenosine 3',5'-cyclic monophosphate sodium salt (8-CPT) was added to the inoculum (A; B) and 6 dpi (C; D). The addition of 8-CPT restored the ability of the mutant to penetrate the ECL. In grey is the auto-fluorescence of the plant. Other abbreviations: IC, infection cushion; LA, lobate appressorium; M, mycelia; RH, runner hyphae; T, trichome. Scale bar: 20 µm. (TIF) [file pone.0091135.s006.tif]
