# Supplementary material for: The Adenylyl Cyclase Plays a Regulatory Role in the Morphogenetic Switch from Vegetative to Pathogenic Lifestyle of Fusarium graminearum on Wheat
Source: PLoS One. 2014 Mar 6;9(3):e91135. doi: 10.1371/journal.pone.0091135 (PMC3946419; doi:10.1371/journal.pone.0091135)
Supplement: Table S1 — Primers used in this study. (DOC) [file pone.0091135.s007.doc]

| **Primers for generation of the gene replacement construct for *Fgac1* (FGSG_01234), the complementation vector p*Fgac1* , the Southern probe and for diagnostic PCRs. Small letters indicate overlapping sequence parts.** | |
| --- | --- |
| **No.**  (see also fig. S1) | **Sequence (5’→ 3’)** |
| 1 | ggccccccctcgaggtcgacggtatcgatccaggttgaaagcgccatgac |
| 2 | gagggcaaaggaatagagtagatgccggacggactgacaagcgatg |
| 3 | gcttccaagcggagcaggctcgacgtattggacagcgtctggactctttgg |
| 4 | gctctagaactagtggatcccccgggctgctcttgctctgtattgctccc |
| 5 | GAGTCACATCCATCAAACCCA |
| 6 | AGACCTTCAATGCCTCTTCC |
| 7 | GCTTACGACTCGAGATGCTTTGT |
| 8 | CCTCTTCTTCTTCGCTGCTGCTAA |
| 9 | ggccccccctcgaggtcgacggtatcgatTGGGTTACGGCATAGAGGCTG |
| 10 | GAAGGTGGTCGGCATAGTGGAAGT |
| 11 | TCCTCCTCCGCTACGACGCCTTCA |
| 12 | ACTTGCTGTAATAACCGCTTCTGC |
| 13 | CTCACGCCTATGGACACTACC |
| 14 | CACAGCACCAGGGCCAAGC |
| 15 | CCTTATGATTGGAATTGGAACCTC |
| 16 | GCTGCTAAGGCTATTCACCGG |
| 17 | TGCGCCTGGAACCGAACTGTGTA |
| 18 | acatgagcatgccctgcccctgagcggccTCAGAATCTTGAGGCGTGTCA |
| 19 | cccgaatcgggaatgcggctctagagtagGCAAGCAGCCCTCGCACCACG |
| 20 | cagcccgggggatccactagttctagagcGCATCGGGGCCAGCACCAAAC |
