# Supplementary material for: The Adenylyl Cyclase Plays a Regulatory Role in the Morphogenetic Switch from Vegetative to Pathogenic Lifestyle of Fusarium graminearum on Wheat
Source: PLoS One. 2014 Mar 6;9(3):e91135. doi: 10.1371/journal.pone.0091135 (PMC3946419; doi:10.1371/journal.pone.0091135)
Supplement: Table S2 — Summary of phenotypes of adenylyl cyclase mutants in other plant fungal pathogens. (DOCX) [file pone.0091135.s008.docx]

|  | **Summary of phenotypes of adenylyl cyclase mutants in other plant fungal pathogens** | | | | | | | | |
| --- | --- | --- | --- | --- | --- | --- | --- | --- | --- |
| **Organism** | **Pathogenicity** | **Appressoria formation** | **Hyphal growth** | **Asexual sporulation** | **Conidial germination** | **Sexual reproduction** | **Secondary metabolism** | **cAMP level** | **reference** |
| *F. graminearum* | nonpathogenic on wheat, fully pathogenic on maize | absent | reduced | reduced in submersed culture, enhanced on agar plates and wheat floral leafs | not affected | like wild type on carrot agar, absent on wheat straw | DON biosynthesis strongly reduced | reduced on wheat and maize | this study |
| *F. proliferatum* | reduced | not tested | reduced | increased | delayed | reduced | increased bikaverin production | not tested | [3] |
| *F. fujikuroi* | not affected ^a)^ | not tested | reduced ^a); b)^ | reduced ^a)^ | not tested | not tested | inappropiate fusarubin biosynthesis, reduced bikaverin and GA production ^b)^ | not tested | a) [4]  b) [44] |
| *C. neoformans* | nonpathogenic ^a)^ | N/A | wild-type like ^a)^ | not tested | not tested | mating defective ^a)^ |  | not detectable ^b)^ | [1] |
| *M. oryzae* | nonpathogenic | absent | reduced | reduced | reduced | sterile | not tested | not tested | [18] |
| *B. cinerea* | attenuated ^a)^ | N/A | Reduced ^a)^ | reduced during infection ^a)^ | reduced ^b)^ | Absent ^b)^ | reduced ^b)^ | reduced in mycelia, increased in germlings ^b)^ | a) [16]  b) [17] |
| *S. sclerotiorum* | attenuated (on wounded leaves) | absent | altered branching pattern | N/A | N/A | aberrant | not tested | reduced | [2] |
| *U. maydis* | reduced | not tested | constitutively filamentous | N/A | N/A | absent | not tested | not tested | [5] |
| *C. lagenarium* | nonpathogenic | nonfunctional appressoria | reduced | reduced | reduced | not tested | not tested | not tested | [6] |

1. Alspaugh JA, Pukkila-Worley R, Harashima T, Cavallo LM, Funnell D, et al. (2002) Adenylyl cyclase functions downstream of the Galpha protein Gpa1 and controls mating and pathogenicity of *Cryptococcus neoformans*. Eukaryot Cell 1: 75-84.

2. Jurick WM, 2nd, Rollins JA (2007) Deletion of the adenylate cyclase (sac1) gene affects multiple developmental pathways and pathogenicity in *Sclerotinia sclerotiorum*. Fungal Genet Biol 44: 521-530.

3. Kohut G, Oláh B, Ádám AL, García-Martínez J, Hornok L (2010) Adenylyl cyclase regulates heavy metal sensitivity, bikaverin production and plant tissue colonization in *Fusarium proliferatum*. J Basic Microbiol 50: 59-71.

4. García-Martínez J, Ádám AL, Avalos J (2012) Adenylyl cyclase plays a regulatory role in development, stress resistance and secondary metabolism in *Fusarium fujikuroi*. PLoS One 7: e28849.

5. Gold S, Duncan G, Barrett K, Kronstad J (1994) cAMP regulates morphogenesis in the fungal pathogen *Ustilago maydis*. Genes Dev 8: 2805-2816.

6. Yamauchi J, Takayanagi N, Komeda K, Takano Y, Okuno T (2004) cAMP-pKA signaling regulates multiple steps of fungal infection cooperatively with Cmk1 MAP kinase in *Colletotrichum lagenarium*. Mol Plant Microbe Interact 17: 1355-1365.
